# Applying Large Graph Neural Networks to Predict Transition Metal Complex Energies Using the tmQM_wB97MV Data Set

**DOI:** 10.1021/acs.jcim.3c01226

**Published:** 2023-12-04

**Authors:** Aaron G. Garrison, Javier Heras-Domingo, John R. Kitchin, Gabriel dos Passos Gomes, Zachary W. Ulissi, Samuel M. Blau

**Affiliations:** † Department of Chemical Engineering, Carnegie Mellon University, Pittsburgh, Pennsylvania 15213, United States; ‡ Department of Chemistry, Carnegie Mellon University, Pittsburgh, Pennsylvania 15213, United States; § Wilton E. Scott Institute for Energy Innovation, Carnegie Mellon University, Pittsburgh, Pennsylvania 15213, United States; ∥ Lawrence Berkeley National Laboratory, Berkeley, California 94720, United States

## Abstract

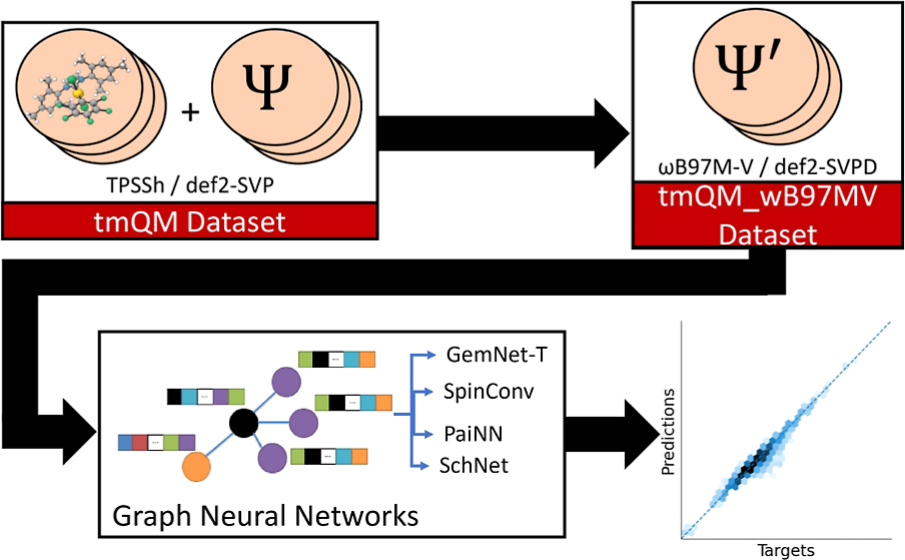

Machine learning (ML) methods have shown promise for discovering novel catalysts but are often restricted to specific chemical domains. Generalizable ML models require large and diverse training data sets, which exist for heterogeneous catalysis but not for homogeneous catalysis. The tmQM data set, which contains properties of 86,665 transition metal complexes calculated at the TPSSh/def2-SVP level of density functional theory (DFT), provided a promising training data set for homogeneous catalyst systems. However, we find that ML models trained on tmQM consistently underpredict the energies of a chemically distinct subset of the data. To address this, we present the tmQM_wB97MV data set, which filters out several structures in tmQM found to be missing hydrogens and recomputes the energies of all other structures at the ωB97M-V/def2-SVPD level of DFT. ML models trained on tmQM_wB97MV show no pattern of consistently incorrect predictions and much lower errors than those trained on tmQM. The ML models tested on tmQM_wB97MV were, from best to worst, GemNet-T > PaiNN ≈ SpinConv > SchNet. Performance consistently improves when using only neutral structures instead of the entire data set. However, while models saturate with only neutral structures, more data continue to improve the models when including charged species, indicating the importance of accurately capturing a range of oxidation states in future data generation and model development. Furthermore, a fine-tuning approach in which weights were initialized from models trained on OC20 led to drastic improvements in model performance, indicating transferability between ML strategies of heterogeneous and homogeneous systems.

## Introduction

Green hydrogen offers a promising alternative to fossil fuels, since it is clean, energy-dense, and does not produce any greenhouse gases when produced or when burned.^1^ However, green hydrogen’s economic viability depends on the discovery of cheaper and more efficient catalysts.^2^ A large challenge in discovering these novel catalysts lies in the enormous chemical space of potential candidates.^3^ Conventional experimental methods require the synthesis, characterization, and assessment of new structures, which is a very time-intensive process that is neither able to completely cover the vast chemical space nor do so in a reasonable amount of time.^4^ To overcome this challenge, computational approaches have been employed since certain material properties, such as electronic energy, can be correlated with catalytic activity. Methods such as density functional theory (DFT) can be used to screen out catalysts that likely have poor performance without the time investment of experimentation.^5^ While useful, a single DFT simulation can still take on the order of days for systems of moderate complexity,^6^ precluding comprehensive high-throughput analysis.

To further reduce the time required to obtain material properties relevant to catalysis, machine learning (ML) models have been used as surrogates for DFT simulations. Once trained, these models can predict species properties on the order of seconds, fast enough to do high-throughput screening.^7^ ML models primarily show promise in screening out poorly performing structures and determining which structures are worth performing full DFT calculations on.^8^ However, since these models have no basis in first-principles chemical physics, they rarely stand alone and can exhibit high errors and poor reliability, especially on systems outside the training domain.^9^ In order for models to have a chance at being generalizable, they require massive and diverse data sets.^3^ Many of the available data sets for catalysis applications are focused on heterogeneous systems, such as OC20 or OC22.^3,10,11^ ML models trained on these large data sets, especially graph neural networks (GNNs), show promise for many applications.^3^ Despite the fact that homogeneous catalysts offer tremendous opportunities for selectivity and tunability that heterogeneous catalysts do not,^12,13^ the field has been lacking similar large-scale, diverse data sets for homogeneous catalysis until the tmQM data set was published in 2020.^11^ Additionally, to the best of our knowledge, no ML potential specifically trained on tmQM has been unveiled; Balcells et al. have since released a modified version of the data set, tmQMg,^14^ which was used to train various graph models.

We took several GNNs that have shown promise on OC20 and OC22 and trained them on tmQM.^3,10^ Surprisingly, these models were consistently unable to predict the energies of a subset of tmQM, even if that data were included in the training set. To address these inconsistencies, we recomputed the energies of structures in tmQM with a higher quality level of electronic structure theory (i.e., single-point calculations), using ωB97M-V/def2-SVPD instead of TPSSh/def2-SVP. Our chosen basis set includes diffuse functions, which should improve energy predictions,^15−17^ particularly given the nature of the electronic structure of the systems studied in tmQM. The ωB97M-V functional is also expected to perform better than TPSSh based on studies of DFT performance on transition metal complexes.^18−21^ The new data set is presented as tmQM_wB97MV and is given alongside a number of ML model benchmarks, learning curves to assess model performances, and a chemical space representation illustrating its improvements. Models trained on tmQM_wB97MV yielded predictions that were consistent with training data, and error metrics improved in all cases.

## Methods

### tmQM ML Model Training

The tmQM data set was designed to provide training and evaluation data for machine learning models that could predict properties of transition metal complexes.^11^ tmQM consists of 86,665 transition metal complexes taken from the Cambridge Structural Database (CSD), whose geometries were optimized using xTB^22^ and given as files listing the atoms in each structure and their Cartesian coordinates. tmQM also contains several properties calculated at the TPSSh/def2-SVP level of theory.^11^ We focus on the total electronic energy of the complexes since, if one can predict the changes in energy associated with a reaction step, they can use that to determine whether that step is favorable and thus if the structure is a good catalyst.

Our first effort to train ML models capable of predicting the energies of transition metal complexes simply used the tmQM data set as originally presented, as molecular structures and associated targets are all that is needed. The data were used without modification, except for translating all the complexes such that their single metal atom was at the origin.

All ML models used in this work are graph neural networks (GNNs) that are publicly available through the Open Catalyst Project (OCP) GitHub repository.^3^ Before these models were trained, several preprocessing steps were carried out. The data presented in tmQM (ASE Atoms objects and, separately, associated properties) were converted into molecular graphs and stored as LMDBs (Lightning Memory-Mapped Databases) using the AtomstoGraphs functionality in OCP. Since the models available in OCP do not incorporate total charge information, and tmQM includes structures with either a −1, 0, or +1 total charge, a subset of tmQM was created containing only neutral structures. This procedure yielded two versions of tmQM: one containing the entirety of tmQM (containing 86,665 structures) and a neutral subset containing 71,173 structures.

Since the electronic energies given by DFT codes are large and those reported for tmQM are nonnormal, additional preprocessing was employed to get the targets close to zero and normally distributed. A reference correction strategy was used, wherein the average energy attributable to each element was computed via a linear regression over the data set and then subtracted from the total energy, which yields a surrogate of the formation energy of the structure. The original distributions of energies, as well as the distributions after reference correction, are shown in the Supporting Information (under “Energy Distributions”). The energies attributed to each element through this strategy are also reported in the Supporting Information (under “Atomic Energies Used for Reference Correction”), for both the entire tmQM data set and the neutral subset. Finally, the data were split between training, testing, and validation sets, using an 80/10/10 split for both all of tmQM and the neutral subset. These splits are included in the supporting code.

After preprocessing, the data were ready to be used in ML workflows. The models’ inputs were the structures, represented as a set of atomic numbers and those atoms’ coordinates. Those atoms composed the graph nodes, and edges were computed on-the-fly during training. The targets were the reference corrected electronic energies.

Data normalization was used during model training with the mean and standard deviation of the targets computed ahead of time. Four GNNs were tested, namely SchNet,^23^ SpinConv,^24^ PaiNN,^25^ and GemNet-T.^26^ Configuration files for all trainings conducted are included in the supporting code. All weights were initialized randomly; i.e., the models were trained from scratch. All models used MAE as their loss metric and were trained using 4 CPU cores, 4 threads, 16 GB of RAM, and one NVIDIA A6000. All four models were trained on both tmQM and the neutral subset of it.

During model training, only training and validation data were used to refine the model. However, models were assessed by their test set performance, obtained using the best-performing checkpoint provided by the model (the weights used during the epoch with the lowest validation MAE) and predicting the test set. In addition to MAE, models were evaluated by energy within threshold (EwT), the percentage of structures for which ML energy predictions were within 1 kcal/mol of the computed DFT energy. Parity plots were also generated, which assess whether the predictions are generally in line with the targets.

Poor predictions on a subset of structures, particularly demonstrated by a consistent region of underprediction across models, motivated the creation of tmQM_wB97MV, to assess whether using a higher quality level of theory would resolve this region of underprediction seen in models trained on tmQM.

### Data Set Generation

Since tmQM provides a large and diverse collection of complexes, its structures and geometries were used as a basis for tmQM_wB97MV. To investigate the impact of the choice of DFT functional and basis set on ML model performance, and to see if we could address the subset of structures whose energies were consistently underpredicted, the energies of the structures in tmQM were recomputed at the ωB97M-V/def2-SVPD level of theory using the Q-Chem electronic structure software^27^ via a high-throughput workflow framework previously reported.^28,29^ In contrast with the TPSSh/def2-SVP level of theory used for tmQM, tmQM_wB97MV’s addition of diffuse functions and the use of a more modern and consistently high-performing functional should improve energy predictions.^15−21^ Despite this increase in quality and cost, only three structures (CSD’s PIFFEP, NASBOA, and LIKBIS) had to be removed due to non-convergence of the DFT code (thanks in large part to the use of on-the-fly error handlers^29^). All of tmQM_wB97MV’s structures can be found in an ASE database linked in the supporting code. While tmQM does include a number of material properties besides the energies, such as the HOMO and LUMO energies, HOMO/LUMO gap, dipole moment, metal natural charge, and polarizability,^11^ since we are interested in catalysis applications and our models are thus designed to take in only the 3D structure of the complexes and output only the corresponding energy, we only recompute and include the energy in tmQM_wB97MV.

Some additional preprocessing was done before finalizing the data set used for training. Since all of the complexes were single metals, the structures’ coordinates were translated such that the metal was at the origin. In addition to this, some structures in the CSD, which were used in tmQM, use implicit hydrogens on some or all of the ligands in the geometry data file presented. This propagated into tmQM, which led to some anomalous structures. 155 such geometries were removed, found by inspecting structures that either lacked hydrogens completely or had a carbon with only one other atom within a 3 Å radius (which was used as a heuristic to find ligands missing hydrogens). An ASE database of these structures is uploaded with the code. We note that additional erroneous structures may remain in the data set. It is difficult to deterministically assess if a structure is invalid, an issue that is compounded by the fact that geometry optimizations conducted with missing hydrogens can lead to structures different from their original representations. Despite this, the energies reported are for the xTB optimized geometries, so besides those data points with erroneous structures carrying little physical meaning, they are not a mismatch between structure and energy, merely a high-energy structure and the corresponding DFT energy.

**Figure 1:**
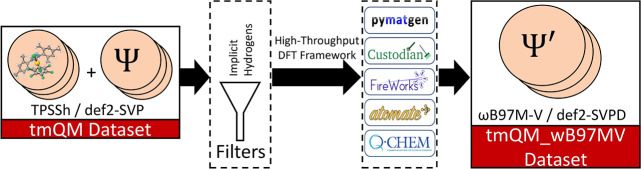
Summary of the methods used to create tmQM_wB97MV from tmQM. First, the transition metal Quantum Mechanics (tmQM) data set,^11^ which contains a diverse set of transition metal complexes, was filtered to remove structures that used implicit hydrogens. Then, the DFT energies of the complexes were recomputed at the ωB97M-V/def2-SVPD level of theory, creating tmQM_wB97MV.

The final product is tmQM_wB97MV, a data set of 86,507 structures taken from tmQM, along with their energies, which have been recomputed at the ωB97M-V/def2-SVPD level of theory. The data are presented as an ASE database file containing, for each structure, an ASE Atoms object that contains the atoms present and their Cartesian coordinates, along with the CSD code, chemical formula, total charge, spin, and electronic energy (in hartree, Ha). While tmQM_wB97MV may still carry some erroneous structures from tmQM, the more accurate energies make it better suited for training ML models that can be used for screening catalysts, as we report below. A summary of the process used to make tmQM_wB97MV is shown in Figure 1.

### tmQM_wB97MV ML Model Training

The ML model training for tmQM_wB97MV follows a methodology very similar to that for tmQM, which was outlined in the “tmQM ML Model Training” section. In summary, the ASE Atoms objects that the structures in tmQM_wB97MV are presented as were converted to graphs and stored in LMDBs using the AtomstoGraphs functionality in OCP. Then, two versions of the data set were created, one containing all 86,507 structures in tmQM_wB97MV, and the other containing the 71,042 neutral structures. The electronic energies were converted to something more akin to formation energy by a reference correction strategy. The original and corrected distributions, as well as the energies used for each element for this method, are included in the Supporting Information (Under “Energy Distributions” and “Atomic Energies Used for Reference Correction”).

After preprocessing, data were split among training, validation, and test sets. These splits were all performed completely randomly. To assess the effects of increasing training data set size on model performance, four different train/val/test splits were used, with 80/10/10, 60/20/20, 40/30/30, and 20/40/40 splits. All of these splits are included in the supporting code. There were eight distinct splits to choose from, stemming from choosing either the entirety of tmQM_wB97MV or just the neutral structures and four splits for each.

The data were then used to train ML models, with the inputs being the atomic numbers and coordinates of each structure (which are the same as the xTB-optimized geometries in tmQM, after centering). Edges were computed on-the-fly during training, and the targets were the reference corrected electronic energies. The same four GNNs as before were trained (SchNet,^23^ SpinConv,^24^ PaiNN,^25^ and GemNet-T^26^). This resulted in 32 models being trained, from eight different splits to choose from and four different models. Configuration files for all training conducted are included in the supporting code. Just as with tmQM, these models were trained from scratch with data normalization and used MAE as their loss metric.

Models were again compared by their test set performance (specifically their MAE, EwT, and parity) using the best-performing checkpoint. tSNE plots of the residuals over the chemical space were also used to determine whether there were species that were predicted particularly poorly relative to others.

In addition to training models from scratch, several transfer learning experiments were run for models trained on tmQM_wB97MV. Transfer learning is an approach wherein the models utilize a pretrained checkpoint from a model trained on a different data set, which is often much larger and more diverse than the one of interest.^30^ This allows for much less training data to be required to reach the same accuracy, or conversely, much higher accuracy given the same amount of training data.^31−33^ We utilize OC20 in our transfer learning approaches since it contains a very large amount of DFT data. OC20 consists of heterogeneous catalysts and small adsorbates,^3^ which is fairly different from the large molecular systems considered here. However, fine-tuning a model trained on OC20 has shown tremendous promise for various data sets, including molecular data.^31^ The experiments done in this work used a simple fine-tuning approach, where the ML models’ weights were initialized from models pretrained on OC20,^3^ and the learning rate was decreased by a factor of 10 compared to training from scratch. Much more complex transfer learning approaches are available, which could further improve performance,^31^ but the method we employed is extremely simple and has demonstrated success in various domains.

## Results and Discussion

### tmQM Results

**Figure 2:**
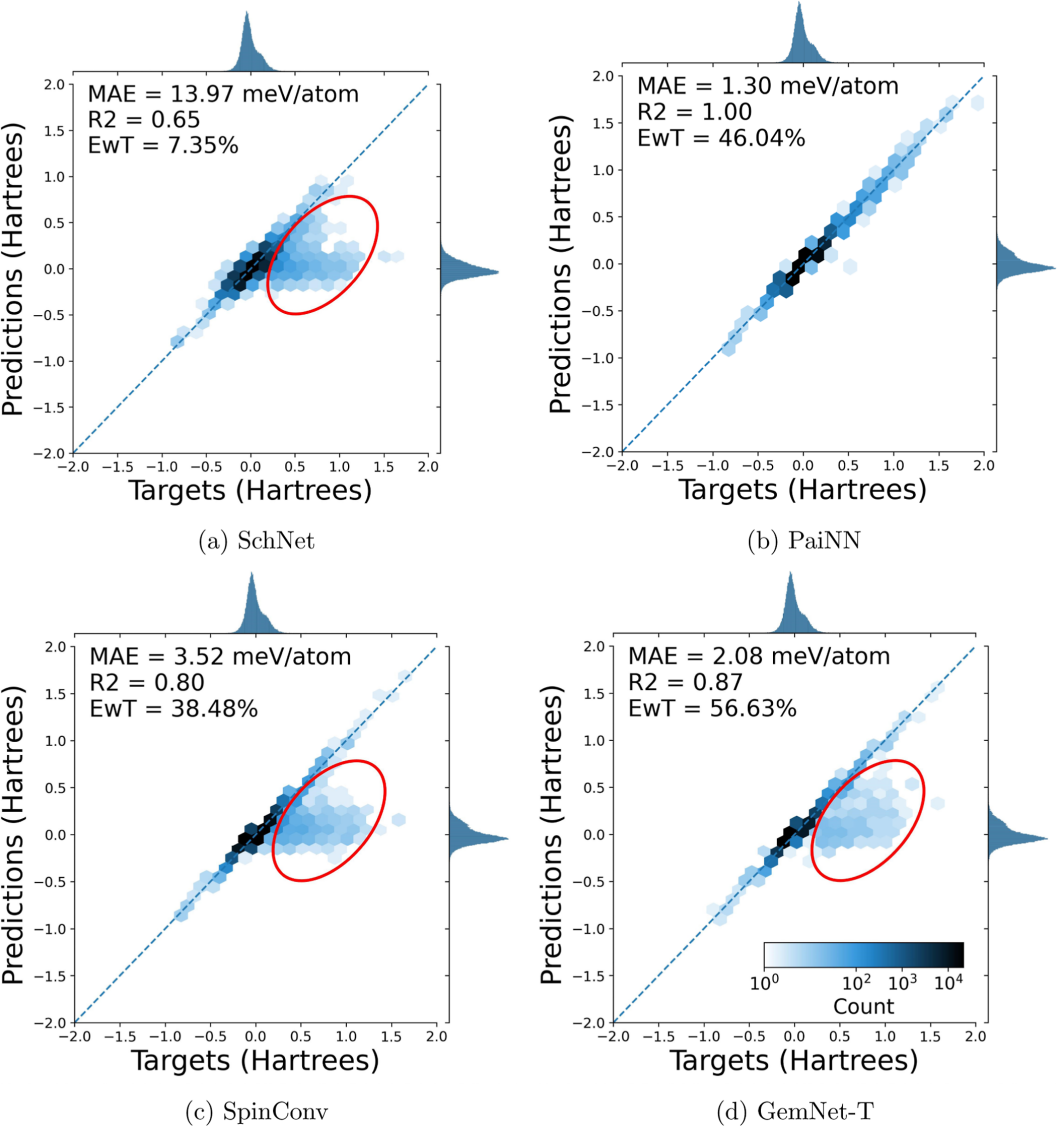
Parity plots of electronic energy predictions vs targets on the train set. The models were all trained with 80/10/10 train/val/test splits on the entirety of the tmQM data set. It is seen that all of the models except PaiNN display a region of underprediction, which is circled in red for visual clarity. In plot 2d, 370 (of 69,333) structures have errors greater than 0.1 hartree.

**Figure 3:**
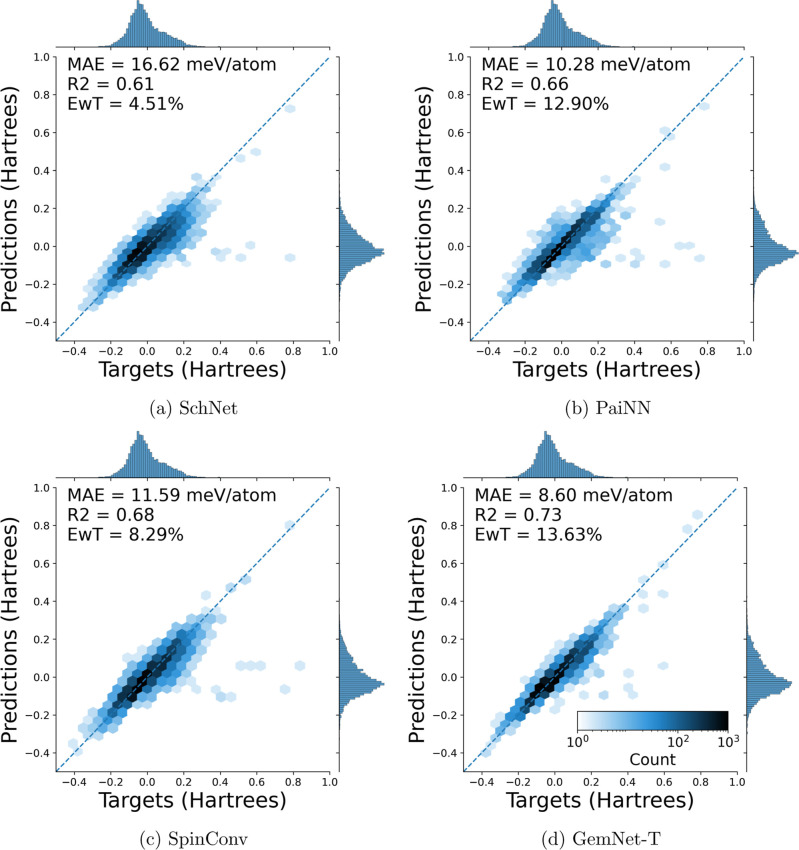
Parity plots of electronic energy predictions vs targets on the test set. The models were all trained with 80/10/10 train/val/test splits on the entirety of the tmQM data set. It is seen that all models display a region of underprediction.

Training on tmQM showed that a subset of the data was consistently predicted very poorly, regardless of the hyperparameters or model used. For an illustration of this, see Figure 2a–d. These figures show parity plots, plotting predictions versus targets, for the training set. If the model were perfect, then all predictions would exactly match the targets and the points would lie on the *y* = *x* line. This behavior is somewhat expected since the predictions are on the training set, which the models were explicitly trained on. However, a subset of the data deviates substantially from the parity line, indicating that the models are not predicting those energies very well. This region is circled in red in Figure 2a,c,d for clarity. While SchNet, SpinConv, and GemNet-T all displayed this region of underprediction in both the training and testing sets, PaiNN differed. PaiNN exhibited excellent performance on the training set, but still showed the region of underprediction on the test set, indicative of overfitting. The test set parity plots can be seen in Figure 3a–d.

One might ask if the region of underprediction highlighted in Figure 2a,c,d is correlated with the 155 removed structures or some other confounding variable, such as charge, since the GNNs trained do not incorporate charge data. To test this, we trained models on the neutral subset of tmQM, and saw that this region of underprediction persists even when charged structures are removed, which is shown in the Supporting Information (Under “tmQM Test Parity Plots”). Additionally, we reconstructed several of the parity plots for models trained on tmQM, with the 155 structures that were removed in tmQM_wB97MV excluded from both the parity plot and the summary statistics. We see that this exclusion has a minimal effect on the summary statistics or the shape of the plot. This is shown in the Supporting Information (under “Effects of Removed Structures on tmQM Statistics”). We thus conclude that the region of underprediction is not due to the 155 removed structures nor charged species and is a legitimate concern.

The persistence of the underpredicted region even on the training set demonstrated that the model was unable to predict the energies of data on which it was explicitly trained, which is indicative of an underlying issue with the DFT data. We later found that these poorly predicted structures were primarily the iron-group metals, namely, Fe, Ru, and Os, which we will discuss more later. This pattern motivated the creation of tmQM_wB97MV, as we hypothesized that adding diffuse basis functions and employing the consistently high-performing ωB97M-V functional would yield more reliable DFT data, which would avoid the observed region of underprediction.

We note that in the generation of tmQM (and also for tmQM_wB97MV), nothing in the DFT data itself is indicative of anything being inconsistent or questionable. The single-point calculations converge, and none of the information in the output signals erroneous data. However, when we train multiple ML models that have been shown to be capable of predicting the energies of various chemistries and they are consistently unable to predict the energies of a subset of the data (even when that data are included in the training set), questions may be raised as to whether those energies are consistent, i.e., if there are issues with the underlying data. While the ML model may just be unable to learn those energies, we can think of it also as a diagnostic tool; the ML model may be able to show inconsistencies in data that would otherwise be unnoticeable. We then want to see if these models are able to predict the energies of the entire data set given energies at a different level of theory that is expected to better capture the chemistry at hand, motivating the generation of tmQM_wB97MV. If so, we have strong evidence that the original data were inconsistent, and the new calculations are an improvement. If not, then studying those structures whose energies cannot be fit may provide valuable insights into either the DFT or the ML models. Either way, ML acts as a useful tool for identifying the quality of data and offers insights beyond what a human observer would be able to come to.

### Data Set Information

Since tmQM_wB97MV contains the same structures as tmQM, the two are essentially identical in terms of the chemical space covered. Both data sets contain only single metal center coordination complexes, the center metal being one of the 30 transition metals in the third, fourth, or fifth periods of the third to twelfth groups in the periodic table. Fourteen elements are allowed in the organic ligands, namely H, C, B, Si, N, P, As, O, S, Se, F, Cl, Br, and I.^11^ The smallest structure in the data set consisted of only five atoms, while the largest had 347 atoms. If one is using the entirety of tmQM_wB97MV, the average number of atoms is 66.02, while the neutral-only subset has 66.32 atoms on average. The distributions of energies are included in the Supporting Information (under “Energy Distributions”).

### Model Performance on tmQM_wB97MV

**Figure 4:**
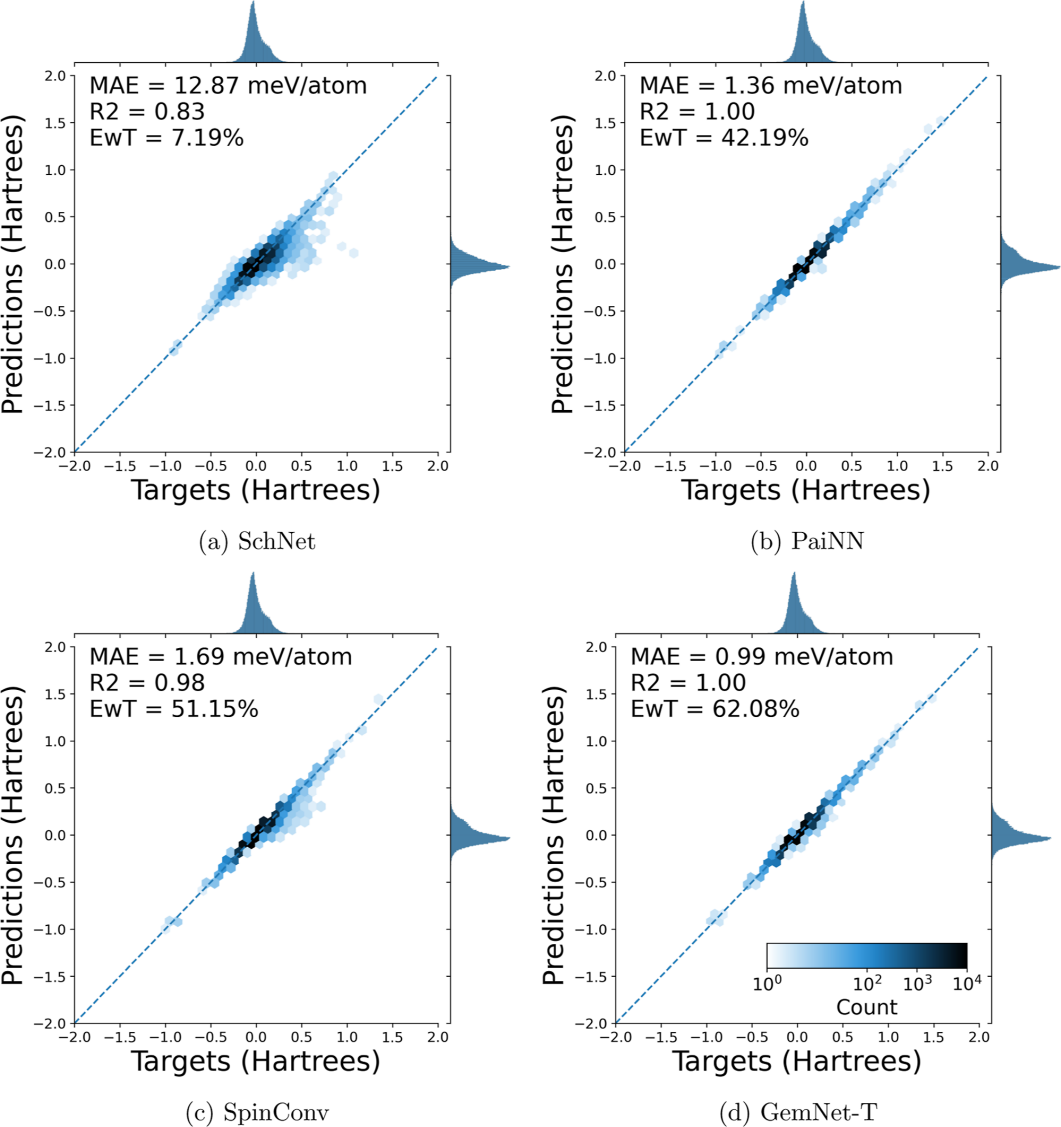
Parity plots of electronic energy predictions versus targets in the training set. Models were trained with 80/10/10 train/val/test splits on the entirety of the tmQM_wB97MV data set were used. Compared to Figure 2a,c,d, it is seen that the regions of poorly predicted structures are no longer present and that evaluation metrics improve.

The first check for tmQM_wB97MV was to see whether the odd predictions seen in Figure 2a,c,d persisted with the improved functional and basis set. Plots comparable to those, i.e., parity plots for predictions on the training data for models trained on 80% of the entire tmQM_wB97MV data set, are shown in Figure 4a–d. From these plots, it is seen that, compared to tmQM, models with the same hyperparameters typically have a better MAE, an improved *R*^2^, and a higher EwT. Most importantly, the region of underprediction, which was present when training on tmQM, is no longer present when using tmQM_wB97MV. With the exception of PaiNN, all models had improved MAE and *R*^2^ values when moving from training on tmQM to training on tmQM_wB97MV.

**Figure 5:**
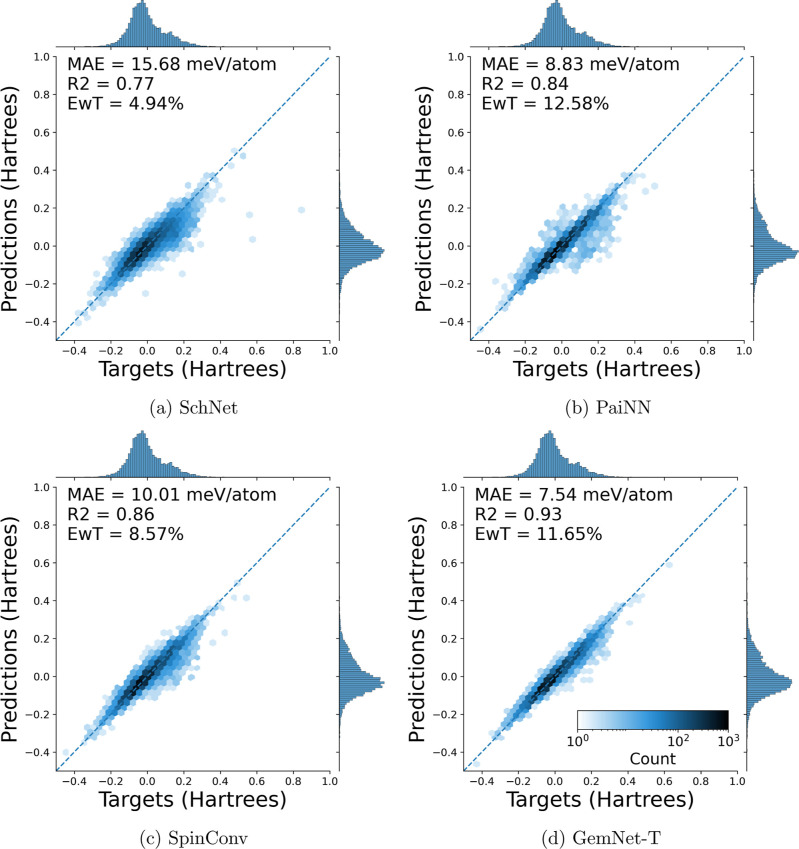
Parity plots of electronic energy predictions versus targets on the test set. The models were all trained with 80/10/10 train/val/test splits on the entirety of the tmQM_wB97MV data set. In general, GemNet-T performs better than both PaiNN and SpinConv, which perform better than SchNet.

These same trends of improved MAE, *R*^2^, and EwT are seen on parity plots for predictions of the testing data. Some examples of these plots can be seen in Figure 5a–d. These show parity plots for the test set of four models trained on 80% of the entirety of tmQM_wB97MV. The rest of the parity plots can be found in the Supporting Information (under “tmQM_wB97MV Test Parity Plots”).

No model displays a significant deviation from parity, and there are no systematic errors in any region of energies. This confirms the models’ applicability on the tmQM_wB97MV data set and that the original level of theory employed for DFT calculations on tmQM was insufficient to consistently describe the electronic structure of all complexes.

After ensuring that the models did not have regions of underpredictions, we moved to benchmark several GNNs on tmQM_wB97MV. Models were mainly compared by MAE, which describes the average error of the model’s predictions; these provide information about how well the model predicts the energies of transition metal complexes in tmQM_wB97MV. The test set MAEs are the primary values of interest since, although the structures in the test set were randomly split from tmQM_wB97MV, the model does not see them during training. Therefore, the predictions of the model on structures in the test set will be more representative of the generalizable performance of the model. MAEs are reported in units of meV/atom. These are scaled by the number of atoms in the structure to account for the fact that energy is an extensive property. Tables of the MAE for all 32 models trained on tmQM_wB97MV are included in the Supporting Information (under “tmQM_wB97MV MAE and EwT Tables”).

In addition to MAE, EwT was also compared between models. EwT gives the percentage of structures that have predicted energies within 1 kcal/mol of the computed DFT energy, providing an indicator of how many structures have ML predictions that provide an acceptable approximation of the DFT ground truth. The test set EwTs for the 16 models trained on all of tmQM_wB97MV are shown in Table 1. The data for the neutral subsets can be found in the Supporting Information (Under “tmQM_wB97MV MAE and EwT Tables”).

**Table 1:** Test Set Energy within Threshold (EwT, %) for Models Trained on all of tmQM_wB97MV^a^

	energy within threshold (EwT, %)			
training %	SchNet	PaiNN	SpinConv	GemNet-T
20	3.2	3.7	5.1	**5.6**
40	4.0	7.3	7.1	**8.9**
60	4.0	10.2	8.8	**11.9**
80	4.9	**12.6**	8.6	11.6

a EwT improves with more training data, and GemNet-T is generally the best-performing model.

**Figure 6:**
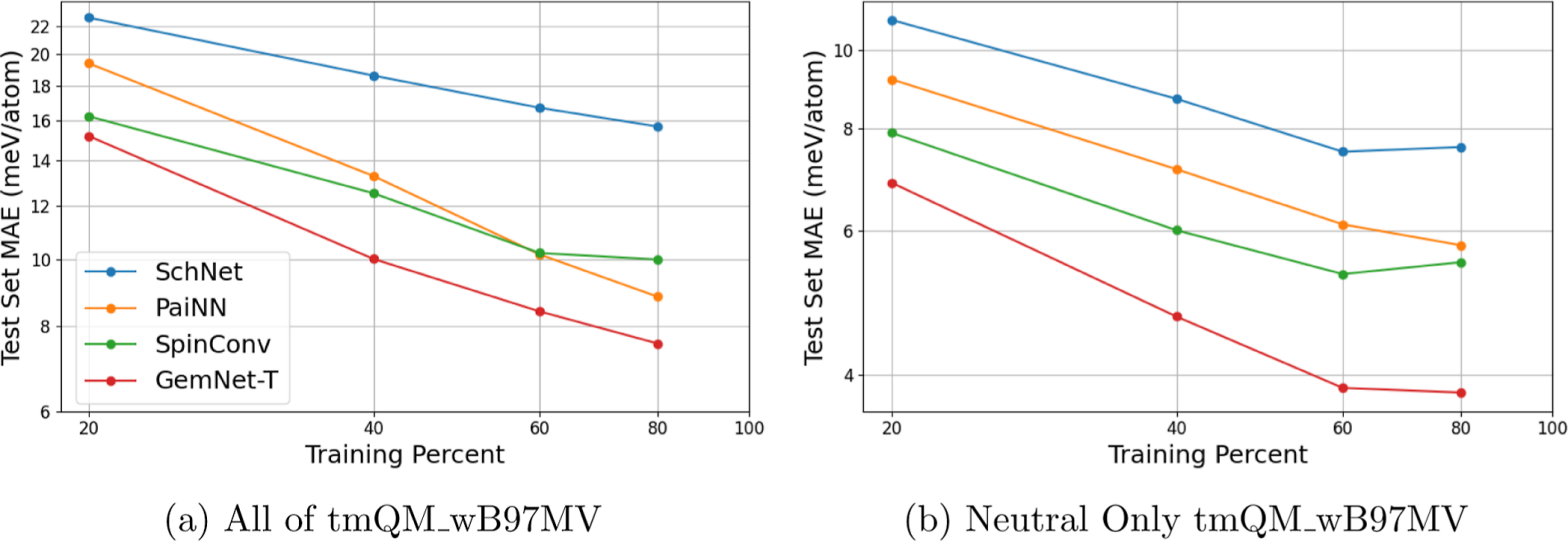
Learning curves for models using all of tmQM_wB97MV (left) and the neutral structures only (right), plotting the test set MAE (in meV/atom) versus the number of training points. The MAE decreases as the training set size increases, with decreasing benefits to more training data. Other models have reported 0.5 meV/atom to be excellent accuracy,^34^ which is not attained by any model trained in this work. Much lower errors are seen when training with only neutral structures, which makes sense since the models do not incorporate charge data. The neutral curves seem to level off at high amounts of data, while models trained with the entire data set do not.

To assess the effects of data set size on ML model performance, models were trained on increasingly large training set sizes, with a focus on how the training set size affected test set MAE for a given model. Learning curves, which are log–log plots of test set MAE versus the amount of training data for both the entirety of tmQM_wB97MV and the neutral subset, are shown in Figure 6a,b.

From Figure 6a,b, we see that the test set MAE decreases with increasing training data for all models, as expected. Additionally, the slope of the GemNet-T curve is steeper than that of SchNet, reinforcing the fact that GemNet-T employs a significantly better equivariant representation of the system. For all models, the rate of decrease for the MAE slows as one increases the training set size further (i.e., the curve starts leveling off), demonstrating that progressively less novel information content is being provided by additional data. This trend is especially pronounced on the learning curves for models trained on the neutral subset, with these curves almost completely leveling off and in some cases actually rising with the last data addition. The neutral-only models do perform much better than those trained on all of tmQM_wB97MV, which is expected since the models used do not incorporate charge information. However, despite their worse overall prediction, we note that the models trained on structures of multiple charges do exhibit a continued decrease in MAE, and for both PaiNN and GemNet-T, the rate of that decrease remains nearly constant up to the full training set size. A model capable of differentiating between and simultaneously learning energies of structures with different charges would almost certainly improve predictions on tmQM_wB97MV. Our observations indicate that the additional chemical information contained in data of multiple charges is critical for continued improvement of model performance with increasing data set size. We further hypothesize that expanding the data set to include open-shell complexes would also greatly increase the available chemical information. Therefore, the combination of a much larger and more diverse data set (in terms of both charge and spin) with a sufficiently discerning and performant model may allow for accurate ML prediction of redox reactions that are common in homogeneous catalysis and models capable of predicting complex reaction pathways more generally.

From the results shown in Figure 6a,b, as well as Table 1, we see that, in general, GemNet-T performs better than both PaiNN and SpinConv, which perform better than SchNet, for both the models trained on the entirety of tmQM_wB97MV and those using only the neutral structures. This broadly correlates with model complexity, age, and how well the models perform on other data sets, such as OC20.^3^ All models tended to perform better when the neutral-only subset of tmQM_wB97MV was used, likely because the lack of charge information in these models made it difficult to predict the energies of charged structures. Finally, as the training set size increased, MAE decreased and EwT increased for all models, which is expected. Training time also increased with the training set size. In general, from smallest to largest training times, the order was SchNet < PaiNN < GemNet-T < SpinConv.

**Figure 7:**
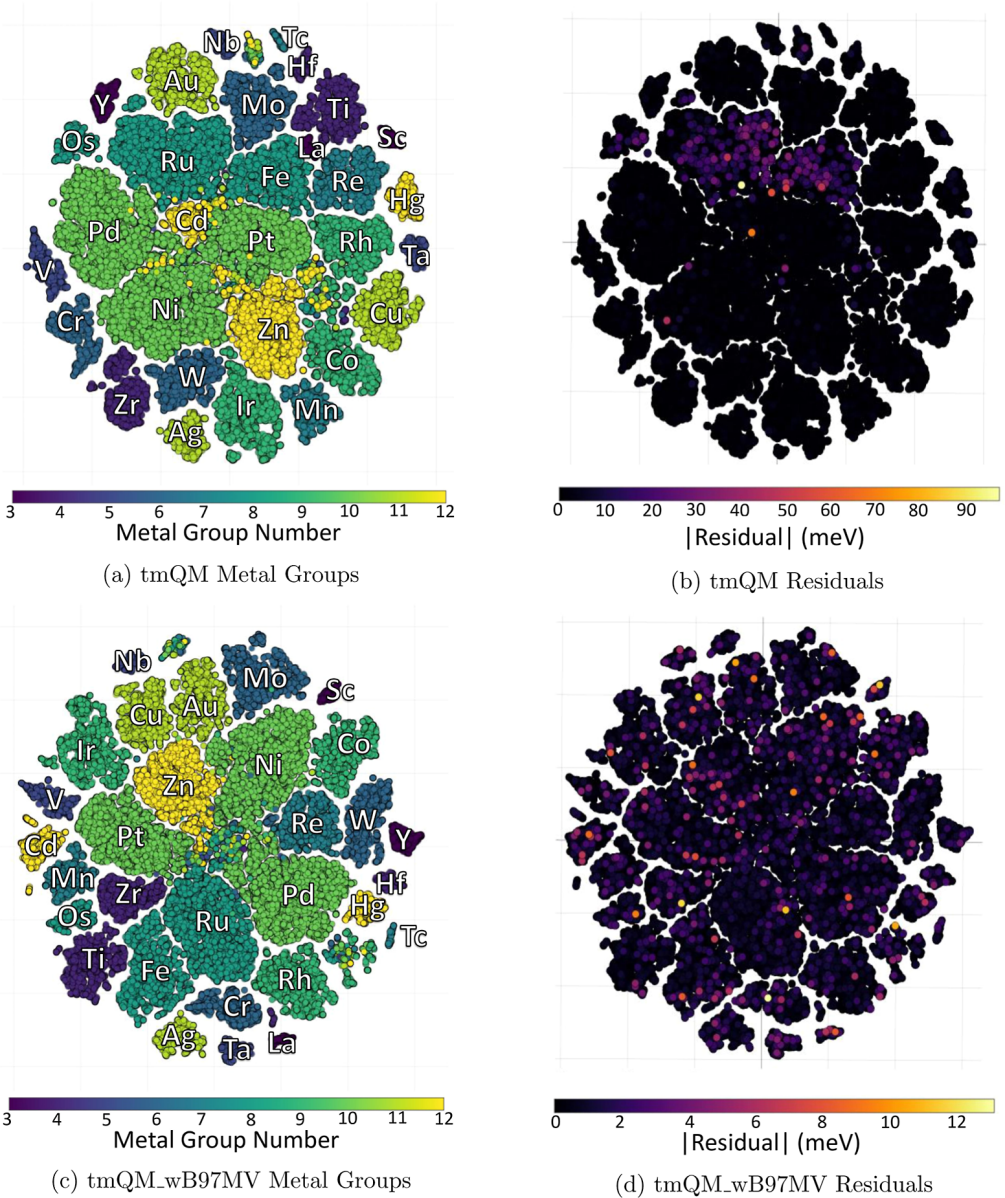
tSNE mappings of the SOAP parameters (after dimensionality reduction with PCA) for the entirety of tmQM and tmQM_wB97MV. Points closer to one another in space are generally more similar in chemical composition, and each island generally corresponds to a specific metal center. This is highlighted on the left, where the homogeneity of colors among islands shows the clustering based on the metal center. On the right, the heatmap colors give the absolute value of the residuals for a GemNet-T model trained on 80% of all tmQM or all tmQM_wB97MV. Each of the bright spots is a single data point, not a cluster of them.

To visualize the data set, structures were characterized using SOAP parameters, which give a quantitative way to determine the similarity between structures.^35^ Then, these parameters were condensed into two dimensions using a tSNE map (after dimensionality reduction with PCA), creating a plot where structures closer to one another were more similar in chemical composition. These studies were done using a combination of the ASAP^36^ and chemiscope^37^ software packages. A tSNE map for tmQM is shown in Figure 7a, and one for tmQM_wB97MV is shown in Figure 7c. The color represents the group of the metal center. Each island in the plot generally corresponds to a metal, as seen in the color clustering.

After these mappings were created, ML models were used to predict energies for the entire data set and then the absolute residuals were used as the heatmap colors (the figures shown use a GemNet-T model trained on 80% of the respective data set). These are shown in Figure 7b for tmQM and Figure 7d for tmQM_wB97MV. To make the residuals more visible, the points were plotted by the absolute value of their residuals on the *z*-axis and then projected back into two dimensions to ensure that the points with more error are on the top of the plot. Figure 7b shows that the iron-group metals were predicted particularly poorly for a model trained on tmQM, while Figure 7d shows that there are no metals that GemNet-T predicts particularly poorly compared to the rest when trained on tmQM_wB97MV (note the substantially different scale). This lack of error clustering, as well as generally lower error, indicates that the models are more consistent and generalizable when trained on tmQM_wB97MV compared to when trained on tmQM.

### tmQM_wB97MV Fine-Tuning Results

**Figure 8:**
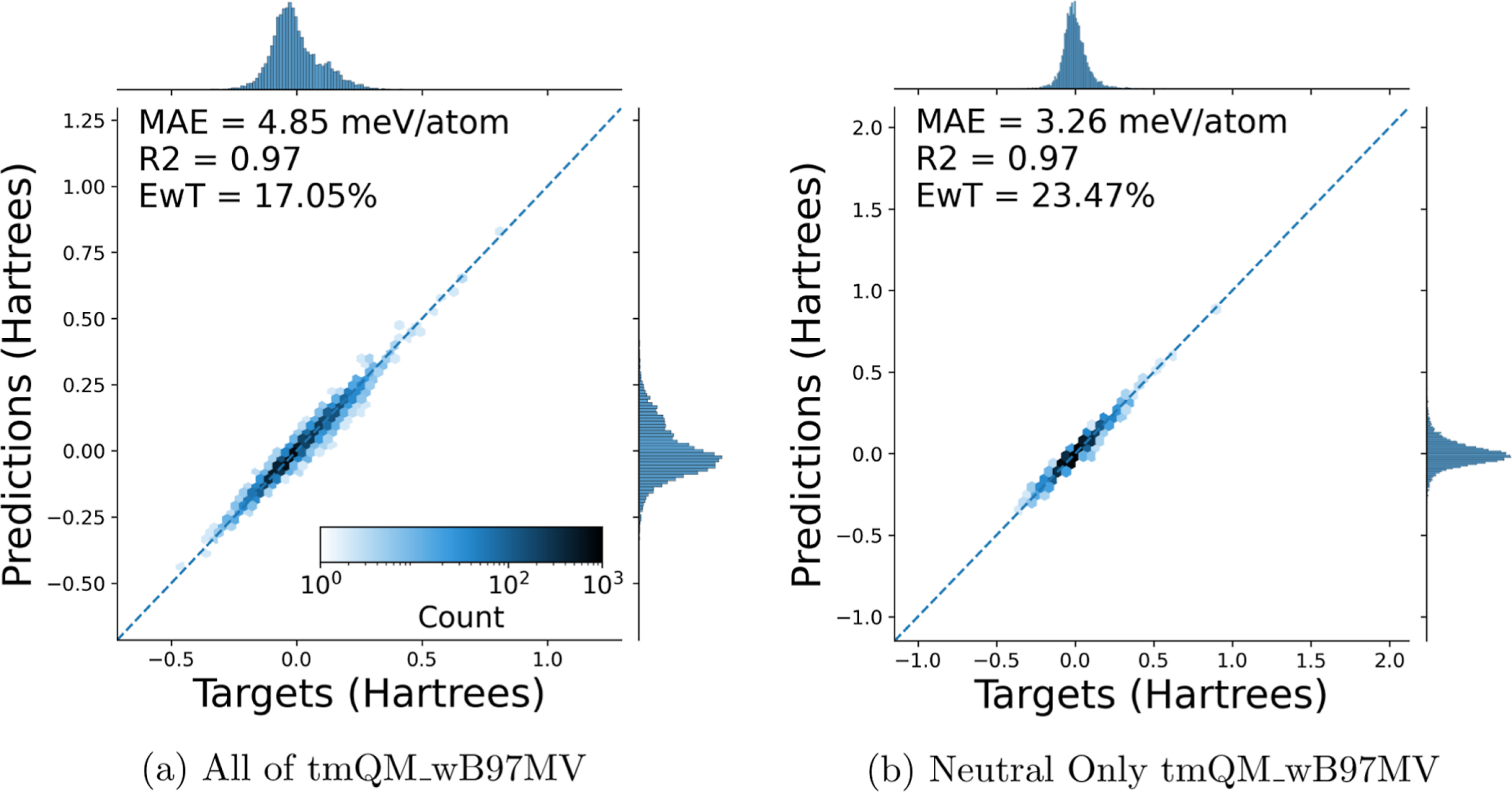
Parity plots of electronic energy predictions vs targets on the test set. A GemNet-T model initialized from a checkpoint trained on the OC20 data set was fine-tuned on 80/10/10 train/val/test splits of the entirety of the tmQM_wB97MV data set (left) and the neutral subset of it (right). Comparing Figures 5d and 8a, it is seen that all performance metrics improve using a fine-tuning approach instead of training from scratch. Similar conclusions are reached by comparing Figure 8b to the neutral results included in the Supporting Information (under tmQM_wB97MV Test Parity Plots).

In addition to training models from scratch on tmQM_wB97MV, GemNet-T^26^ models were fine-tuned on all of tmQM_wB97MV and the neutral subset of it, using the publicly available checkpoints from OC20^3^ as the starting point. Compared to the models trained from scratch, the fine-tuned models show improvements in performance, with a 35.8% lower MAE on the full data set, and a 14.2% reduction on the neutral subset. Parity plots of the test set predictions for the fine-tuned GemNet-T^26^ models can be seen in Figure 8a,b. Figure 8a shows a model fine-tuned on 80% of all tmQM_wB97MV, which has drastically lower MAE and higher EwT compared to an equivalent model trained from scratch, as shown in Figure 5d. This improvement in model performance (an MAE of 4.85 meV/atom, an *R*^2^ of 0.97, and an EwT of 17.05% compared to 7.54, 0.93, and 11.65 from scratch), attained merely by initializing the weights from those of a model trained on OC20, demonstrates tremendous potential for fine-tuning in this domain. Figure 8b shows a model fine-tuned on 80% of the neutral subset of tmQM_wB97MV and demonstrates much less drastic improvements (an MAE of 3.26 meV/atom, an *R*^2^ of 0.97, and an EwT of 23.47% compared to 3.80, 0.96, and 25.24 from scratch). Despite this, the improvement in both cases by initializing weights from a model trained on OC20 shows both the promise of transfer learning in this domain and how results from heterogeneous catalysis can carry over to homogeneous systems.

## Conclusions

The tmQM_wB97MV data set is presented, which takes structures from tmQM, recomputes their electronic energies at the ωB97M-V/def2-SVPD level of theory, and filters out some of the erroneous structures that are missing hydrogens. Several GNNs benchmarked on this data set show that ML models are able to predict transition metal complex energies fairly well and that their performance improves when trained on tmQM_wB97MV compared to tmQM. In particular, tmQM_wB97MV alleviates the chemically distinct underprediction observed with tmQM. Using a fine-tuning approach from large data sets in heterogeneous catalysis further improves performance, which is promising for the concept of large, generalizable models. Despite improved results with tmQM_wB97MV, the data set as-is will not be sufficient for training ML models capable of screening transition metal complex catalysts because it does not span all species relevant to reactions, where the most glaring omissions are open-shell complexes and reactive intermediates. Additionally, the inability of the ML models trained to account for different charge and spin states will be a significant hindrance in predicting homogeneous catalytic reaction pathways, where redox often plays a central role. Expanding the data set to include open-shell complexes would greatly increase the chemical information contained therein, as would additional charged data. This is supported by the fact that models trained on even a small amount of charged data demonstrate continued improvement of model performance with increasing data set size, instead of leveling off, which occurs when only neutral structures are used. The combination of a much larger and more diverse data set (in terms of both charge and spin and reactive intermediate structures) with a sufficiently discerning and performant model architecture could allow for accurate prediction of complex reaction pathways in homogeneous catalysis. There are two major directions for future work: (1) developing models capable of differentiating between and simultaneously learning energies of structures with different charges and spin states, and (2) building an expanded data set that includes a much wider variety of charge and spin states to enable models trained on such data to be used in catalysis applications. These approaches should seek to demonstrate that, given a large and chemically diverse data set, ML models can generalize across the homogeneous catalyst space.

## Data Availability

The data set and supporting code are available at: https://github.com/ulissigroup/tmQM_wB97MV, which includes: ASE Atoms representations of the removed structures, as well as the versions of tmQM and tmQM_wB97MV that were trained on; Files used to generate Figure 7b,d via chemiscope; Configuration files for all models trained, to be used with the OCP repository;^3^ Jupyter notebooks and markdown files explaining how to use the repository; Configuration files, checkpoints, and test set predictions for models fine-tuned from OC20; Predictions of the test set energies for all models trained; Energies used for reference correction for each of the data sets trained on; Scripts used for pre- and postprocessing; Trained checkpoints for the models trained with 80% of the training data on a given data set (Checkpoints for models trained with a lesser percentage are available upon request); and LMDBs containing the data used to train ML models in this work, which also includes the data splits used.

## Supporting Information Available

The supporting information includes: Histograms of the energy distributions for each of the four data sets models were trained on (all of tmQM, the neutral subset of tmQM, all of tmQM_wB97MV, and the neutral subset of tmQM_wB97MV), both before and after reference correction; Tables of the atomic energies used for each of the four data sets models were trained on; MAE and EwT tables for models trained on 20, 40, 60, or 80% of tmQM and the neutral subset of tmQM; Learning curves for models trained on tmQM and the neutral subset of tmQM; Test set parity plots for all 32 models trained on tmQM and the neutral subset of tmQM; Parity plots for a GemNet-T model trained on 80% of tmQM, with the structures that were removed from tmQM to tmQM_wB97MV removed, and a parity plot of only the removed structures; MAE and EwT tables for models trained on 20, 40, 60, or 80% of tmQM_wB97MV and the neutral subset of tmQM_wB97MV; and Test set parity plots for all 32 models trained on tmQM_wB97MV and the neutral subset of tmQM_wB97MV (PDF)

## Notes

Zachary Ulissi is now a Research Scientist at Meta Fundamental AI Research. However, this work was completed during his time at Carnegie Mellon University.
