# Supplementary material for: Applying Large Graph Neural Networks to Predict Transition Metal Complex Energies Using the tmQM_wB97MV Data Set
Source: J Chem Inf Model. 2023 Dec 4;63(24):7642–54. doi: 10.1021/acs.jcim.3c01226 (PMC10751796; doi:10.1021/acs.jcim.3c01226)
Supplement: Supplementary file 1 — ci3c01226_si_001.pdf [file ci3c01226_si_001.pdf]

Supporting Information for

Applying Large Graph Neural Networks to Predict Transition Metal Complex Energies Using the tmQM_wB97MV Dataset

Aaron G. Garrison,[†] Javier Heras-Domingo,[†] John R. Kitchin,[†] Gabriel dos Passos Gomes,[†,‡,¶] Zachary W. Ulissi,[*,†,¶] and Samuel M. Blau[*,§]

†*Department of Chemical Engineering, Carnegie Mellon University, Pittsburgh PA 15213*

‡*Department of Chemistry, Carnegie Mellon University, Pittsburgh PA 15213*

¶*Wilton E. Scott Institute for Energy Innovation, Carnegie Mellon University, Pittsburgh, PA 15213*

§*Lawrence Berkeley National Laboratory, Berkeley CA 94720*

E-mail: zulissi@andrew.cmu.edu; smblau@lbl.gov

Energy Distributions

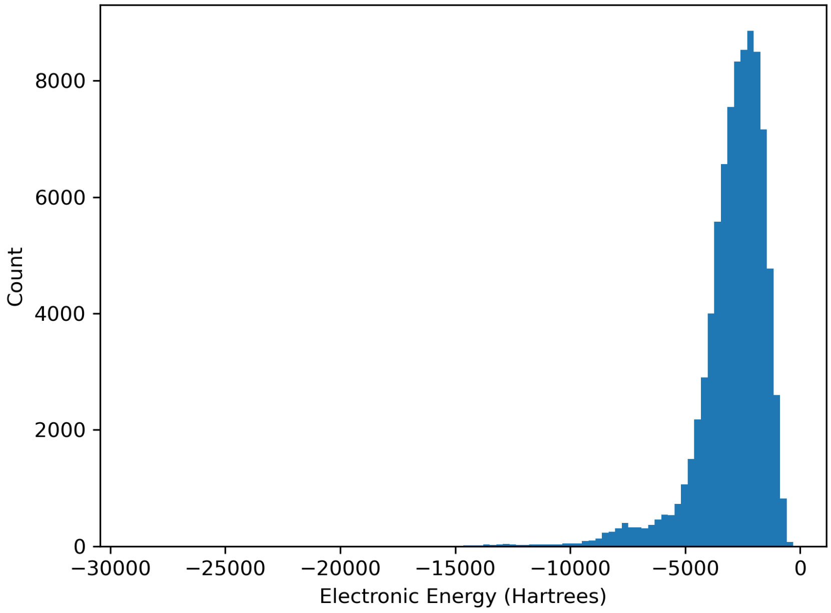

(a) Uncorrected

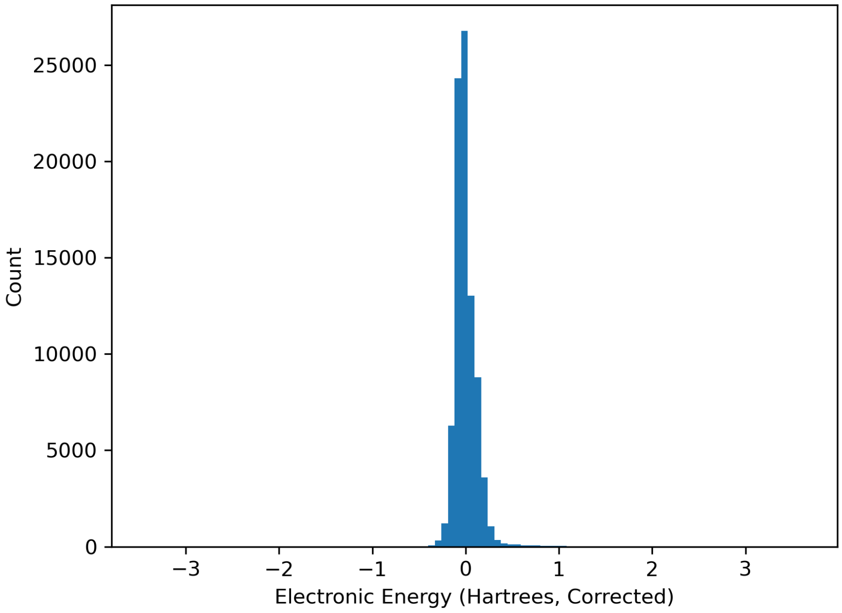

(b) Corrected

Figure 1: Energy distributions of tmQM before preprocessing (top) and after preprocessing (bottom). Both histograms plot the count versus the electronic energy in hartrees.

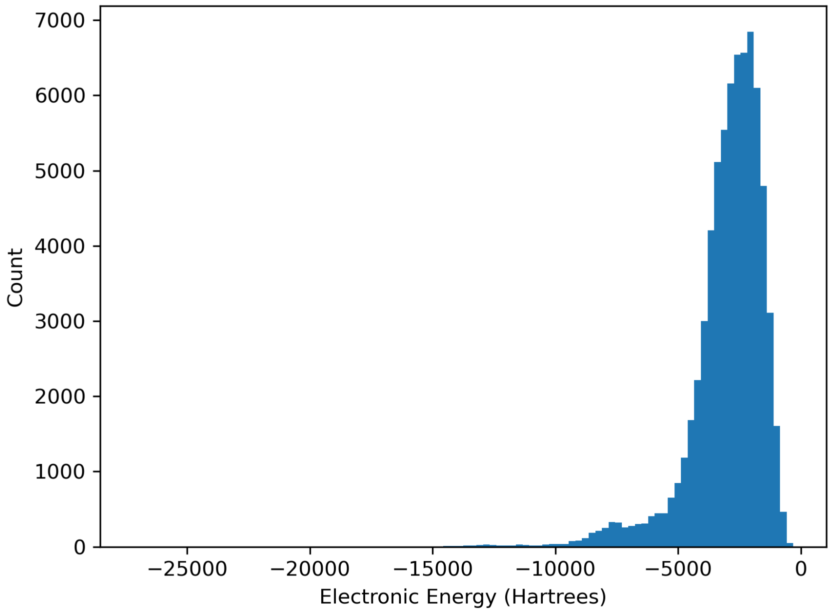

(a) Uncorrected

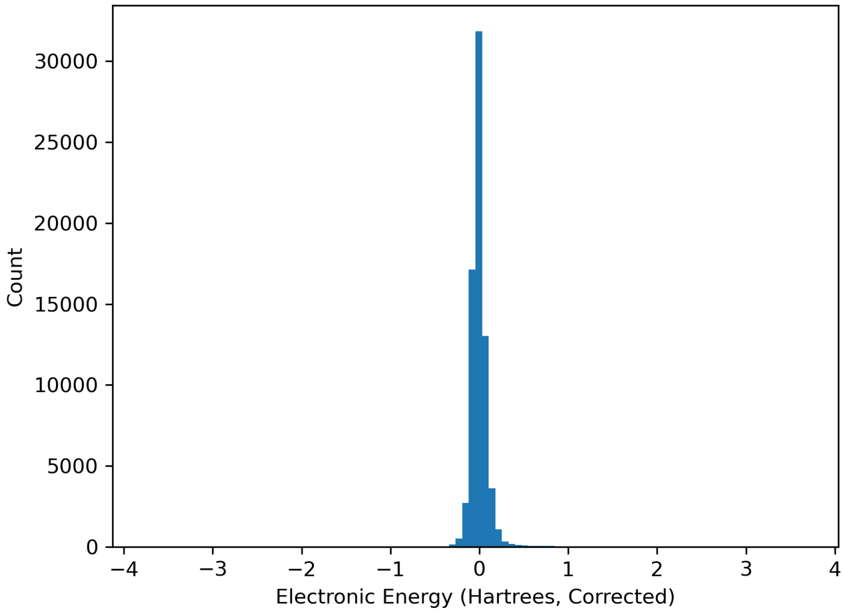

(b) Corrected

Figure 2: Energy distributions of the neutral subset of tmQM before preprocessing (top) and after preprocessing (bottom). Both histograms plot the count versus the electronic energy in hartrees.

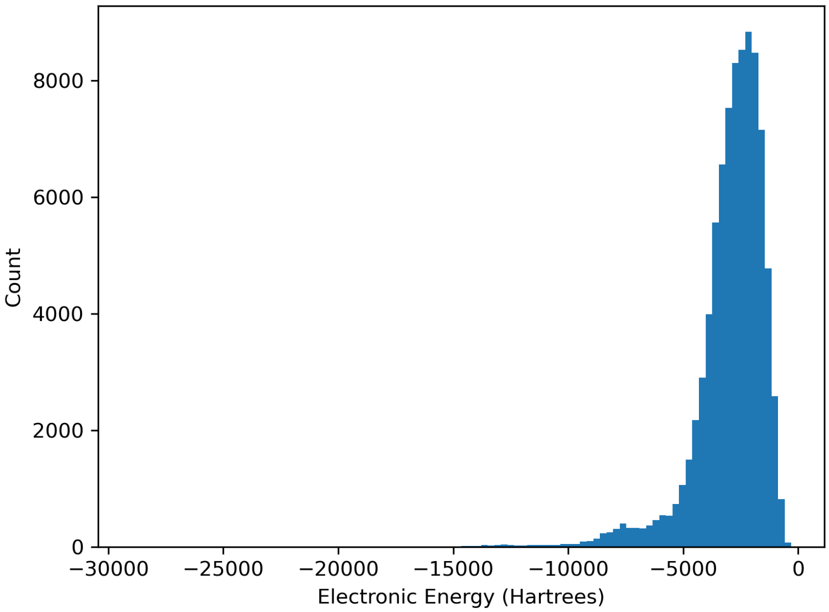

(a) Uncorrected

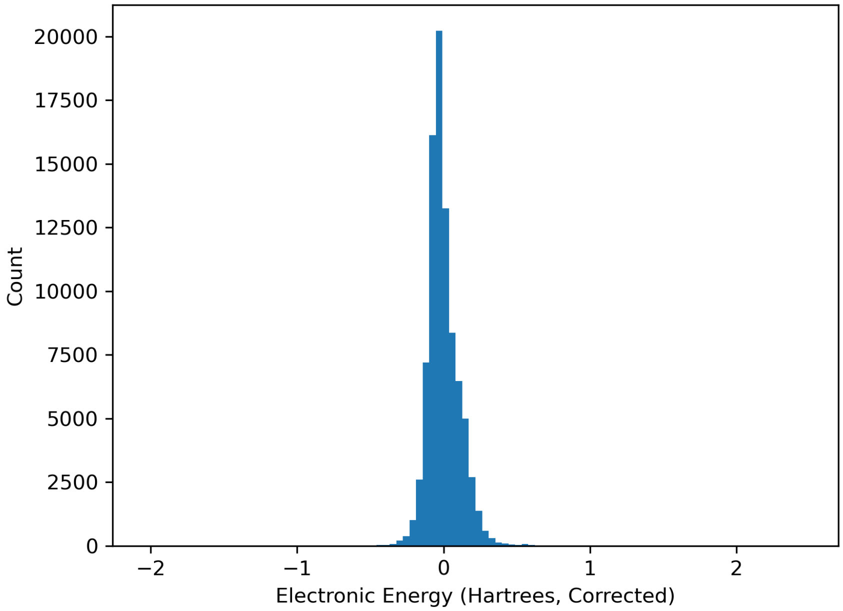

(b) Corrected

Figure 3: Energy distributions of tmQM_wB97MV before preprocessing (top) and after preprocessing (bottom). Both histograms plot the count versus the electronic energy in hartrees.

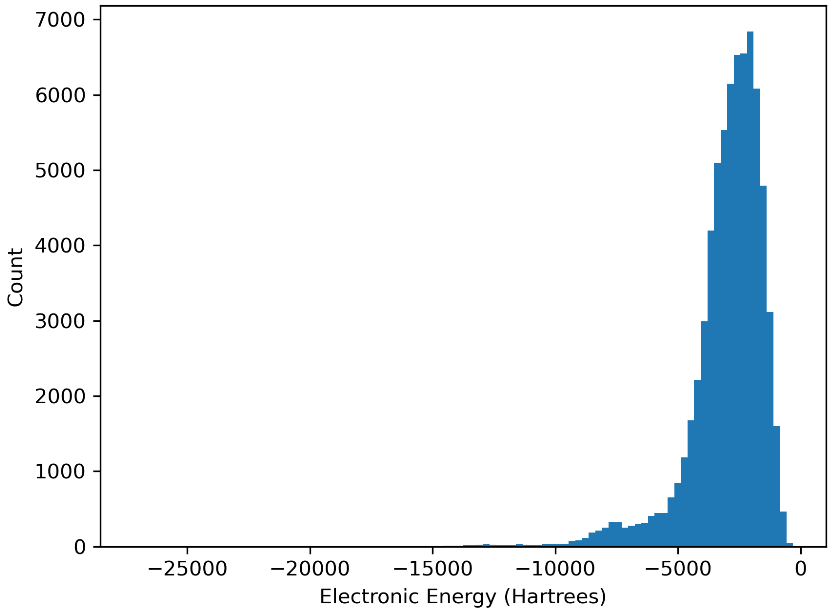

(a) Uncorrected

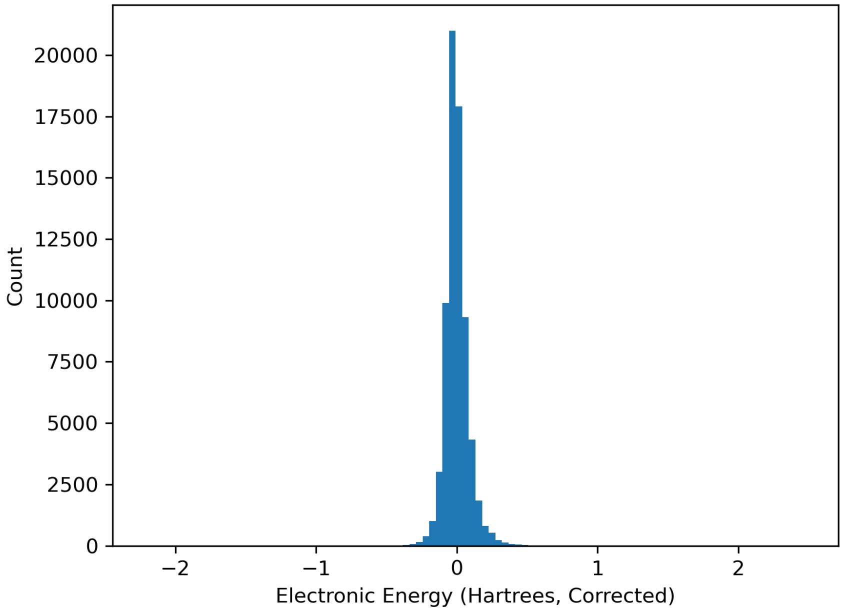

(b) Corrected

Figure 4: Energy distributions of the neutral subset of tmQM_wB97MV before preprocessing (top) and after preprocessing (bottom). Both histograms plot the count versus the electronic energy in hartrees.

Atomic Energies Used for Reference Correction

Table 1: Atomic energies used for reference correction on the entire tmQM dataset.

Atomic Number	Energy (Hartrees)
1	-0.60
5	-24.87
6	-38.09
7	-54.71
8	-75.17
9	-99.76
14	-289.48
15	-341.28
16	-398.10
17	-460.11
21	-760.70
22	-849.48
23	-943.98
24	-1044.38
25	-1150.86
26	-1263.54
27	-1382.58
28	-1508.14
29	-1640.24
30	-1779.16
33	-2235.57
34	-2401.23
⋮	⋮

Atomic Number	Energy (Hartrees)
⋮	⋮
35	-2573.81
39	-38.39
40	-47.13
41	-57.05
42	-68.24
43	-80.81
44	-94.84
45	-110.49
46	-127.82
47	-146.78
48	-167.62
53	-297.67
57	-31.61
72	-48.10
73	-57.07
74	-67.14
75	-78.35
76	-90.63
77	-104.34
78	-119.31
79	-135.63
80	-153.32

Table 2: Atomic energies used for reference correction on the neutral subset of the tmQM dataset.

Atomic Number	Energy (Hartrees)
1	-0.60
5	-24.86
6	-38.09
7	-54.72
8	-75.17
9	-99.76
14	-289.47
15	-341.29
16	-398.10
17	-460.10
21	-760.71
22	-849.50
23	-943.99
24	-1044.39
25	-1150.92
26	-1263.57
27	-1382.63
28	-1508.17
29	-1640.31
30	-1779.18
33	-2235.57
34	-2401.23
⋮	⋮

Atomic Number	Energy (Hartrees)
⋮	⋮
35	-2573.80
39	-38.41
40	-47.15
41	-57.07
42	-68.27
43	-80.87
44	-94.90
45	-110.55
46	-127.85
47	-146.86
48	-167.65
53	-297.65
57	-31.63
72	-48.11
73	-57.09
74	-67.16
75	-78.39
76	-90.70
77	-104.40
78	-119.34
79	-135.68
80	-153.35

Table 3: Atomic energies used for reference correction on the entire tmQM_wB97MV dataset.

Atomic Number	Energy (Hartrees)
1	-0.60
5	-24.85
6	-38.07
7	-54.70
8	-75.16
9	-99.75
14	-289.44
15	-341.23
16	-398.05
17	-460.07
21	-760.62
22	-849.37
23	-943.85
24	-1044.23
25	-1150.73
26	-1263.44
27	-1382.45
28	-1508.03
29	-1640.13
30	-1779.07
33	-2235.49
34	-2401.16
⋮	⋮
⋮	⋮
35	-2573.75
39	-38.31
40	-47.03
41	-56.93
42	-68.11
43	-80.69
44	-94.77
45	-110.41
46	-127.76
47	-146.75
48	-167.61
53	-297.74
57	-31.49
72	-48.02
73	-56.95
74	-67.01
75	-78.22
76	-90.53
77	-104.24
78	-119.22
79	-135.56
80	-153.30

Table 4: Atomic energies used for reference correction on the neutral subset of the tmQM_wB97MV dataset.

Atomic Number	Energy (Hartrees)
1	-0.60
5	-24.84
6	-38.07
7	-54.70
8	-75.16
9	-99.75
14	-289.43
15	-341.24
16	-398.05
17	-460.06
21	-760.63
22	-849.39
23	-943.85
24	-1044.25
25	-1150.78
26	-1263.47
27	-1382.49
28	-1508.05
29	-1640.20
30	-1779.09
33	-2235.50
34	-2401.16
35	-2573.74
39	-38.33
40	-47.05
41	-56.95
42	-68.14
43	-80.75
44	-94.83
45	-110.47
46	-127.79
47	-146.83
48	-167.64
53	-297.73
57	-31.52
72	-48.03
73	-56.97
74	-67.03
75	-78.27
76	-90.60
77	-104.30
78	-119.25
79	-135.62
80	-153.33

tmQM MAE and EwT Tables

Table 5: Test set Mean Absolute Error (in meV/atom) for all models trained on all of tmQM.

	MAE (meV/atom)			
Training %	SchNet	PaiNN	SpinConv	GemNet-T
20%	23	20	17	**16**
40%	20	15	14	**11**
60%	17	11	11	**9**
80%	17	10	12	**9**

Table 6: Test set Mean Absolute Error (in meV/atom) for models trained on the neutral subset of tmQM.

	MAE (meV/atom)			
Training %	SchNet	PaiNN	Spinconv	GemNet-T
20%	12	10	8	**8**
40%	10	8	8	**6**
60%	9	7	7	**6**
80%	8	7	6	**5**

Table 7: Test set Energy within Threshold (EwT, %) for models trained on the entirety of tmQM.

	Energy within Threshold (EwT, %)			
Training %	SchNet	PaiNN	Spinconv	GemNet-T
20%	3.3	3.7	5.0	**5.8**
40%	3.8	6.6	6.8	**9.6**
60%	4.5	10.2	9.4	**12.7**
80%	4.5	12.9	8.3	**13.6**

Table 8: Test set Energy within Threshold (EwT, %) for models trained on the neutral subset of tmQM.

	Energy within Threshold (EwT, %)			
Training %	SchNet	PaiNN	Spinconv	GemNet-T
20%	6.0	7.7	10.0	**11.0**
40%	8.3	11.0	11.8	**16.2**
60%	9.4	13.5	13.9	**20.1**
80%	10.6	15.3	15.1	**21.3**

tmQM Learning Curves

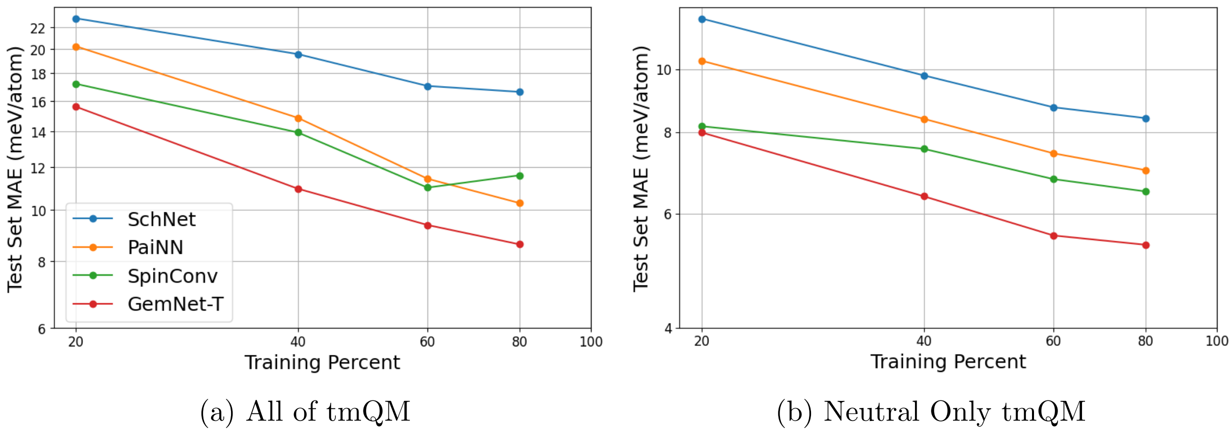

Figure 5: Learning curves for models using all of tmQM (left) and the neutral structures only (right), plotting test set MAE (in meV/atom) versus the percentage of the data used for training.

tmQM Test Parity Plots

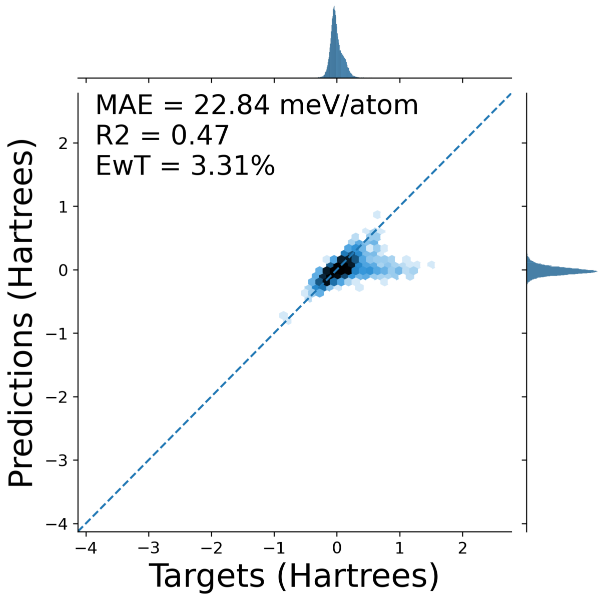

(a) SchNet

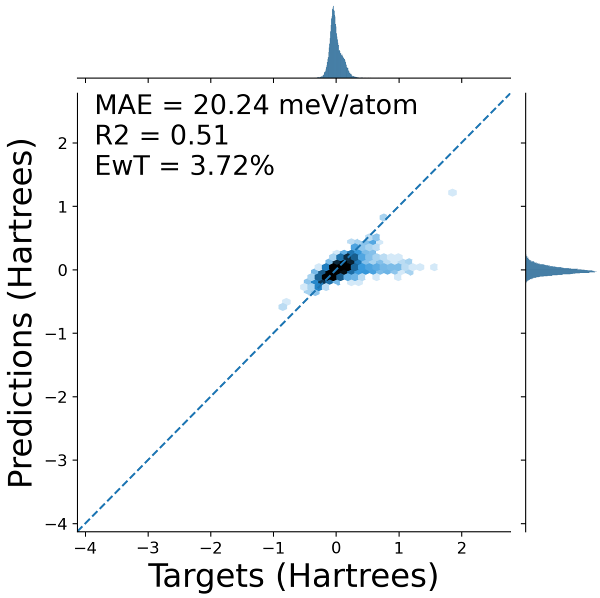

(b) PaiNN

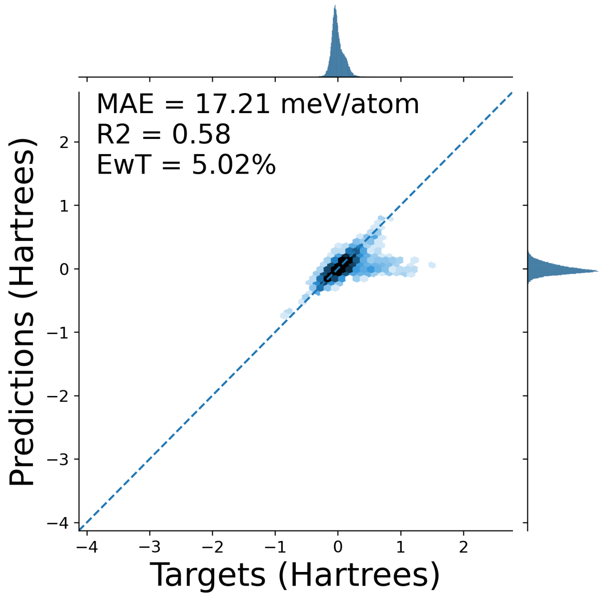

(c) SpinConv

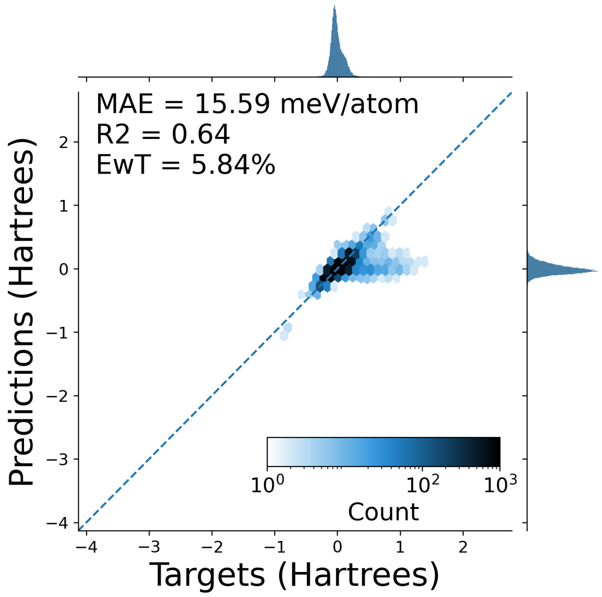

(d) GemNet-T

Figure 6: Parity plots for the test set of models trained on 20% of tmQM.

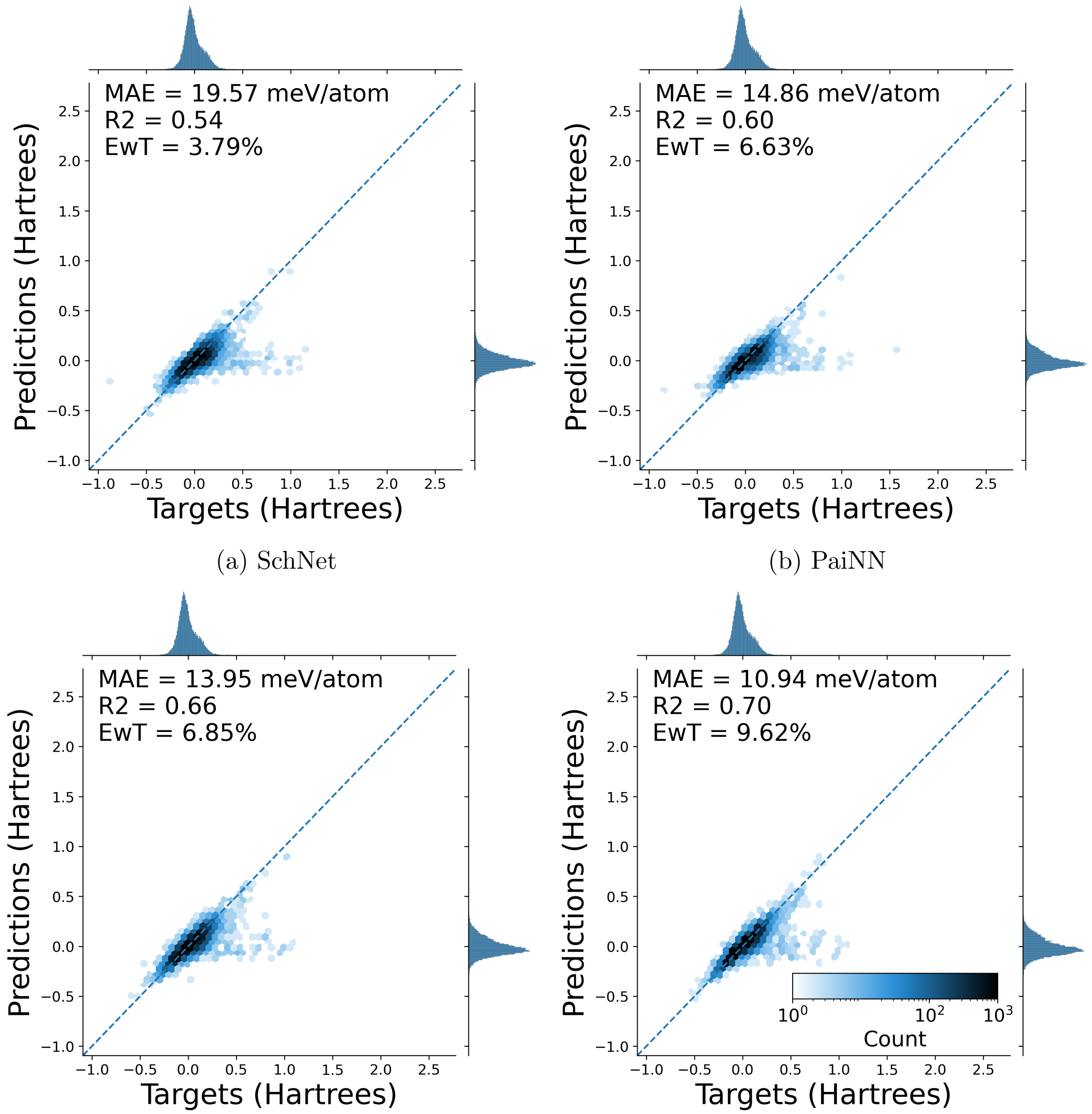

(a) SchNet (b) PaiNN (c) SpinConv (d) GemNet-T

Figure 7: Parity plots for the test set of models trained on 40% of tmQM.

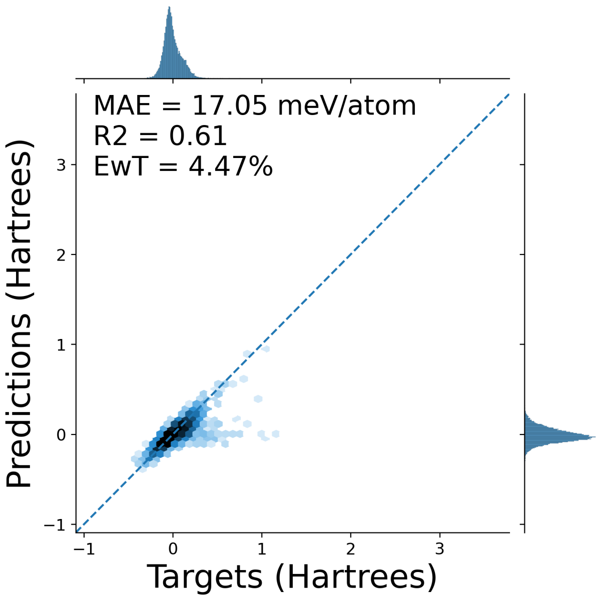

(a) SchNet

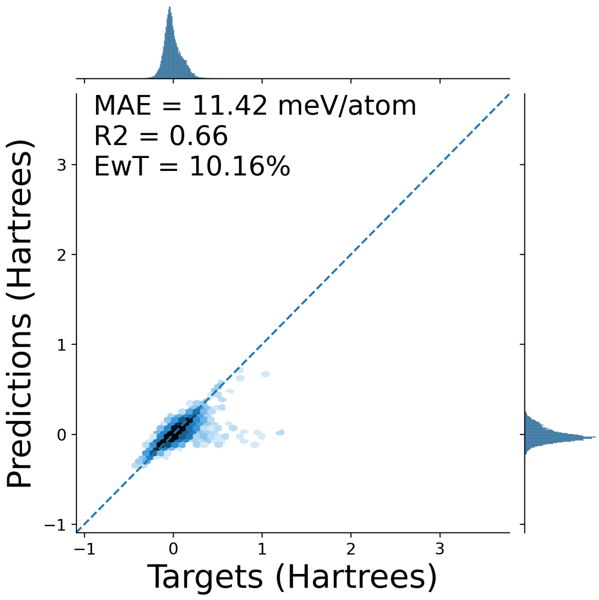

(b) PaiNN

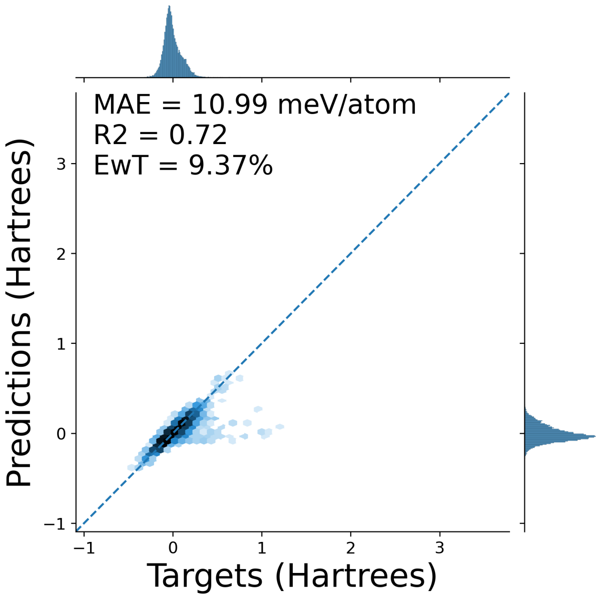

(c) SpinConv

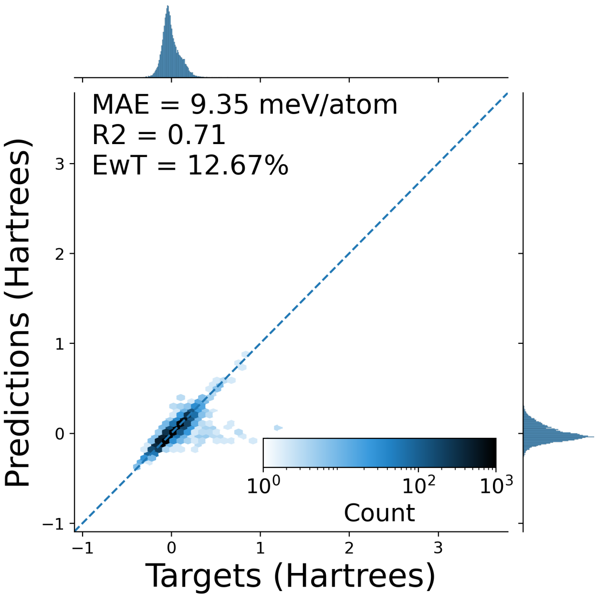

(d) GemNet-T

Figure 8: Parity plots for the test set of models trained on 60% of tmQM.

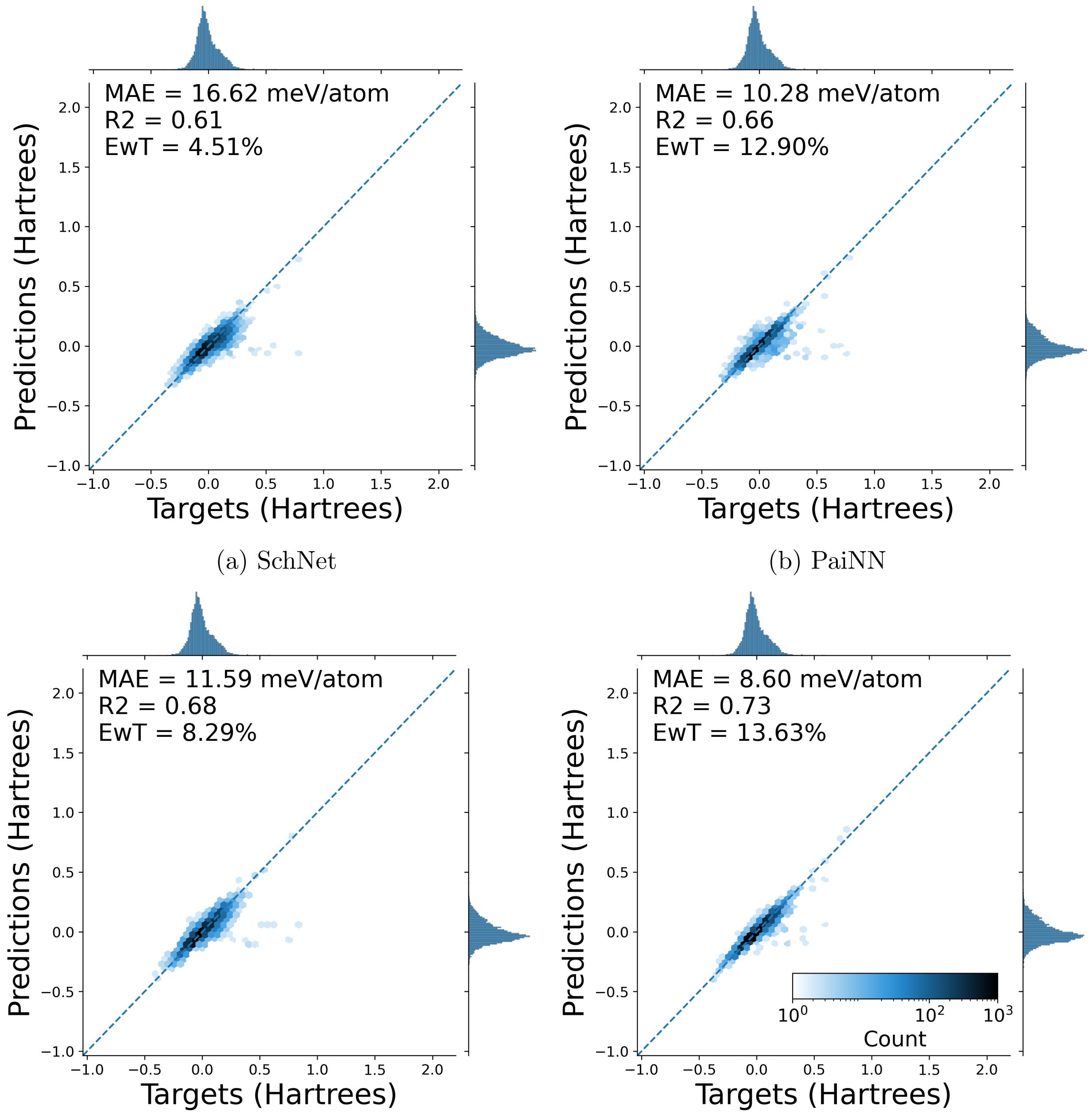

(a) SchNet

(b) PaiNN

(c) SpinConv

(d) GemNet-T

Figure 9: Parity plots for the test set of models trained on 80% of tmQM.

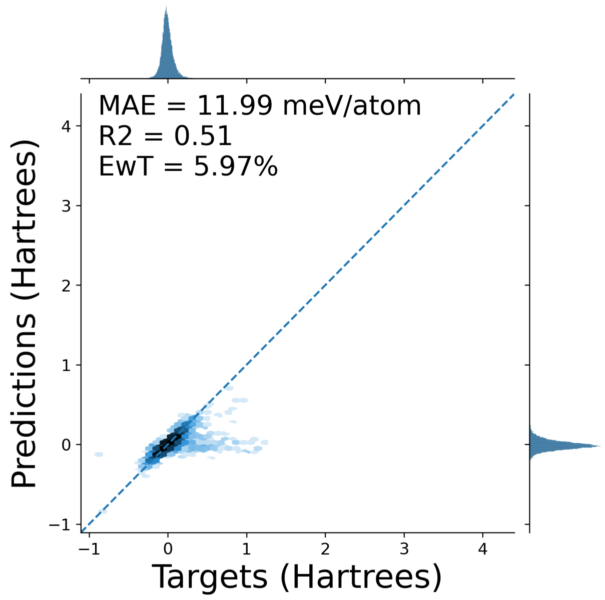

(a) SchNet

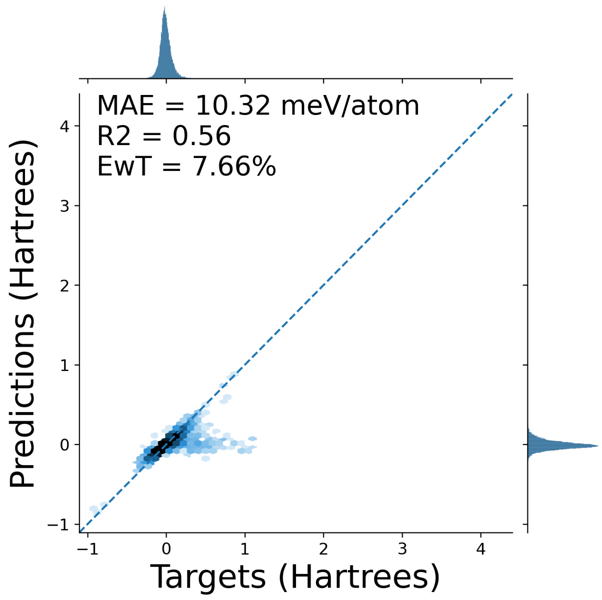

(b) PaiNN

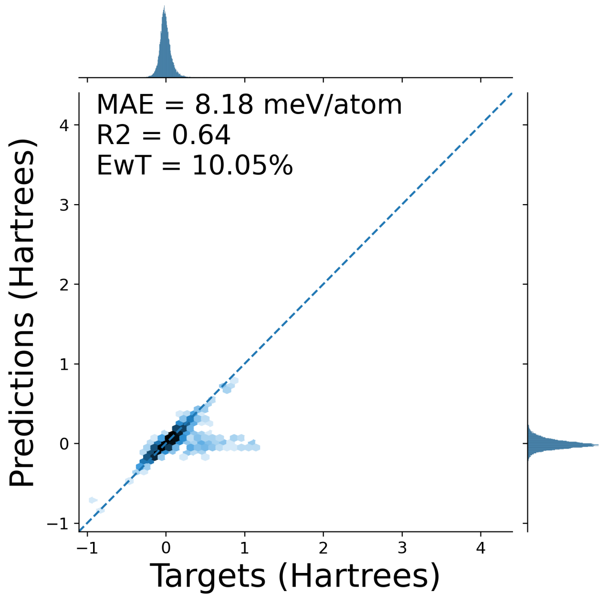

(c) SpinConv

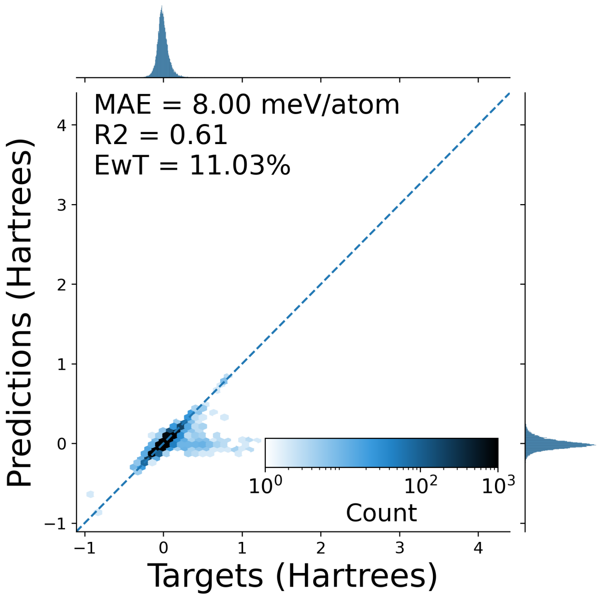

(d) GemNet-T

Figure 10: Parity plots for the test set of models trained on 20% of the neutral subset of tmQM.

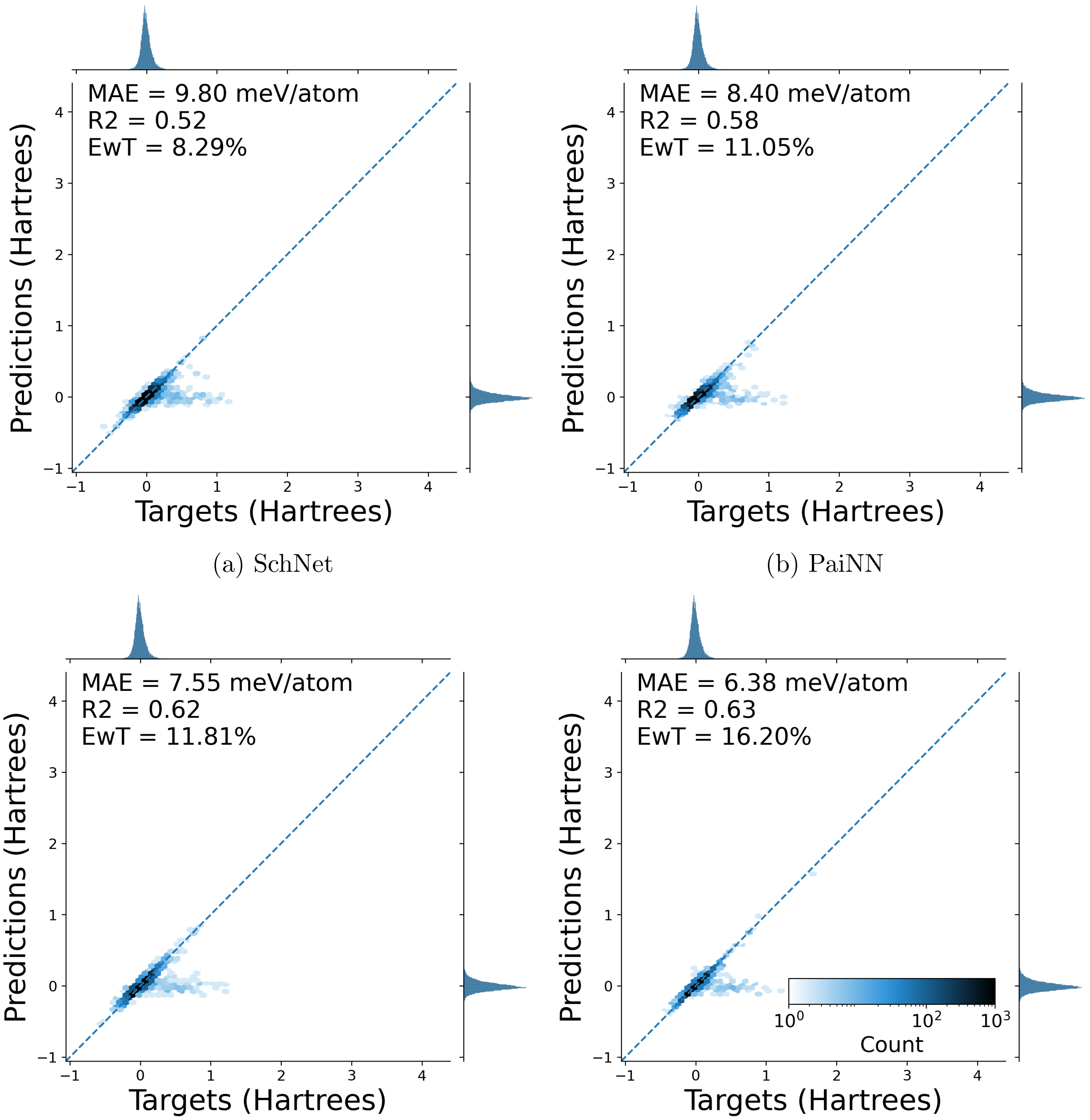

(a) SchNet

(b) PaiNN

(c) SpinConv

(d) GemNet-T

Figure 11: Parity plots for the test set of models trained on 40% of the neutral subset of tmQM.

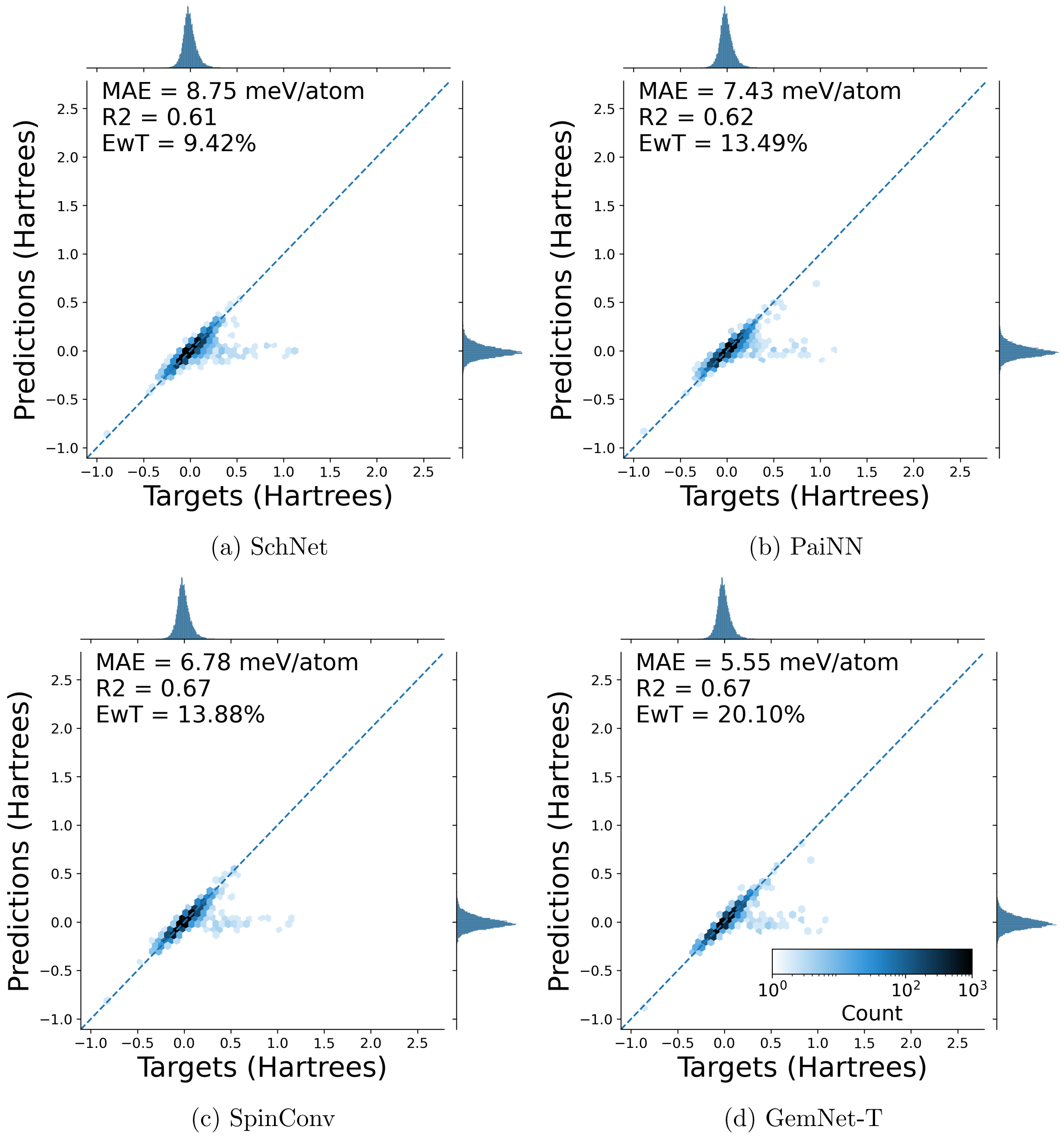

(a) SchNet

(b) PaiNN

(c) SpinConv

(d) GemNet-T

Figure 12: Parity plots for the test set of models trained on 60% of the neutral subset of tmQM.

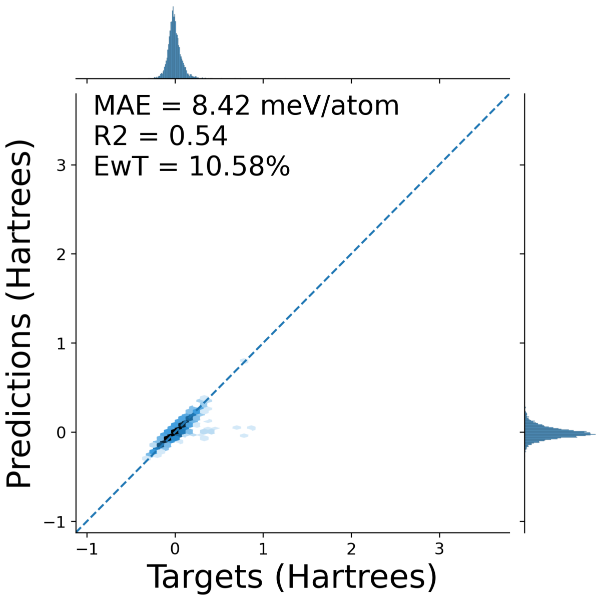

(a) SchNet

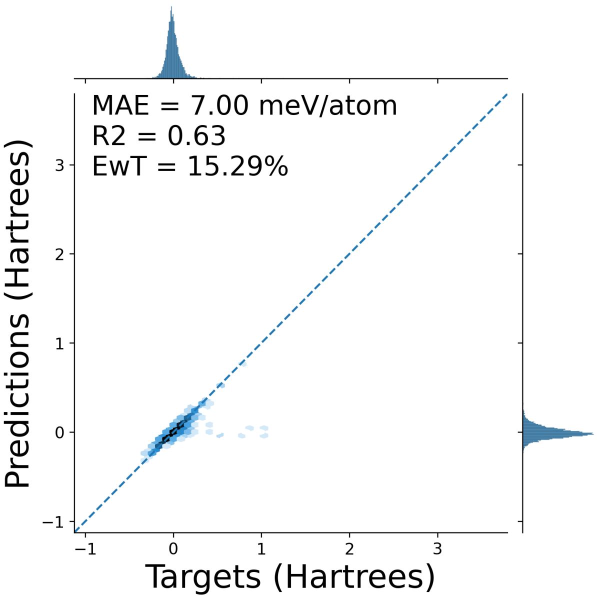

(b) PaiNN

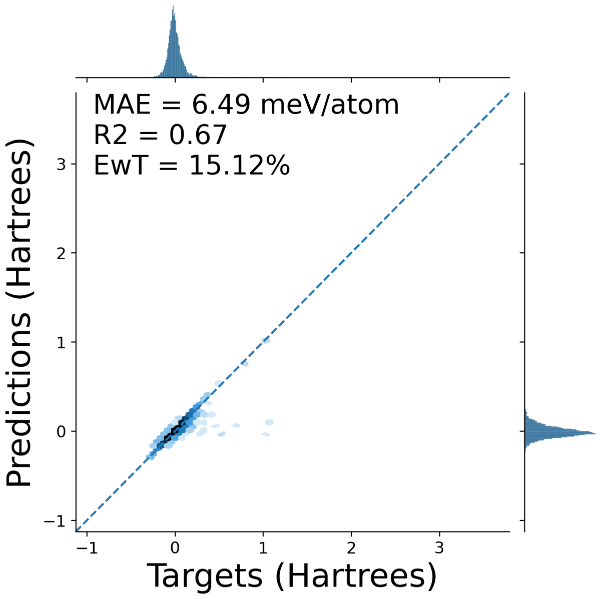

(c) SpinConv

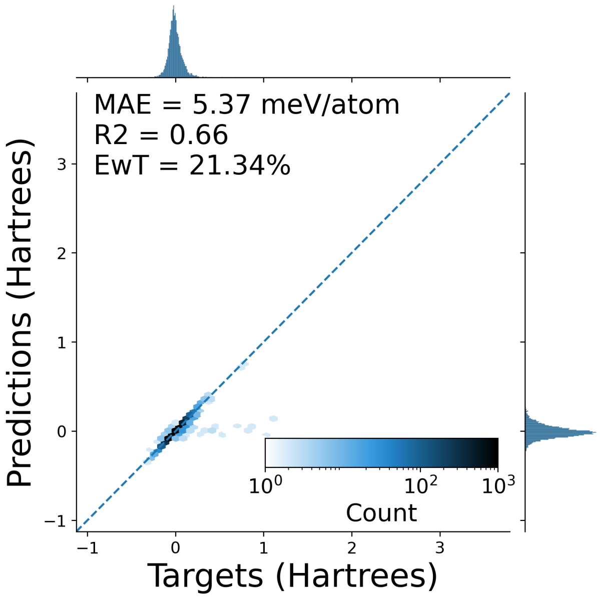

(d) GemNet-T

Figure 13: Parity plots for the test set of models trained on 80% of the neutral subset of tmQM.

Effects of Removed Structures on tmQM Statistics

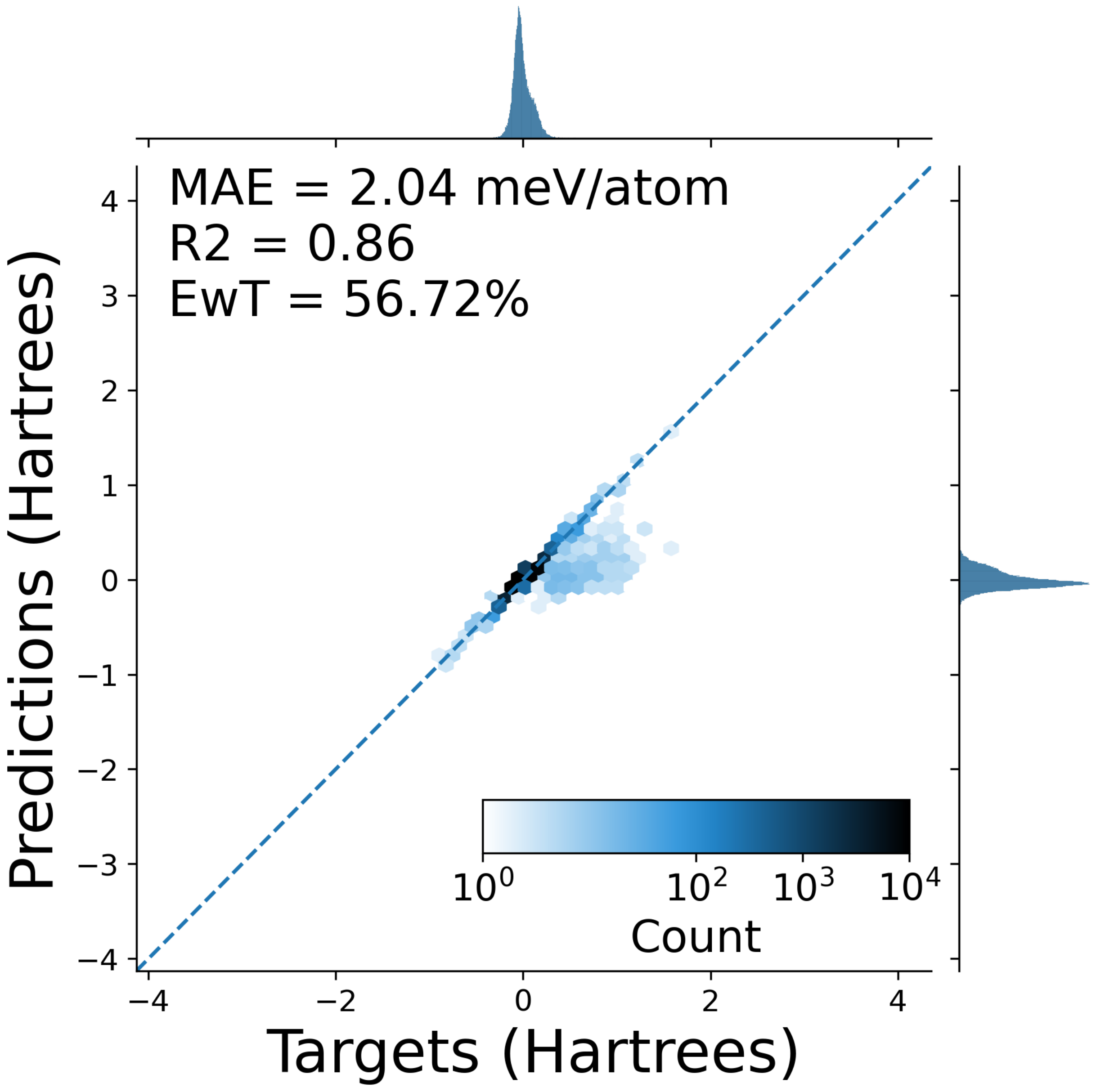

Figure 14: Parity plot for the training set of a GemNet-T model trained on 80% of all of tmQM, with the datapoints corresponding to structures removed from tmQM to tmQM_wB97MV removed. Of the 69,333 structures in the training set, 362 structures had an absolute error of at least 0.1 Hartree, and 172 had errors of at least 0.5 Hartree. 119 structures in this set were structures that were removed when generating tmQM_wB97MV.

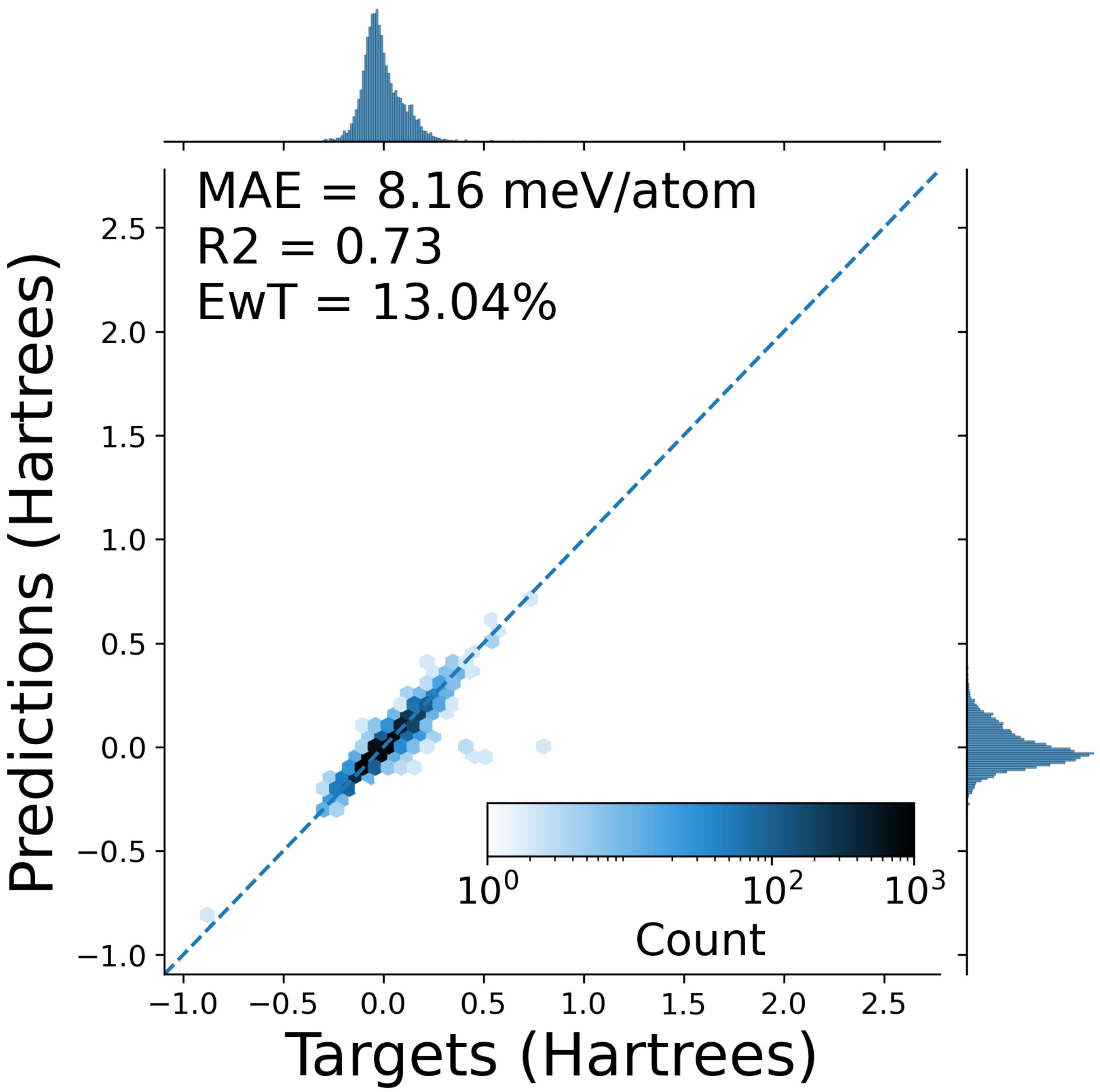

Figure 15: Parity plot for the validation set of a GemNet-T model trained on 80% of all of tmQM, with the datapoints corresponding to structures removed from tmQM to tmQM_wB97MV removed. Of the 8,666 structures in the validation set, 166 structures had an absolute error of at least 0.1 Hartree, and 31 had errors of at least 0.5 Hartree. 13 structures in this set were structures that were removed when generating tmQM_wB97MV.

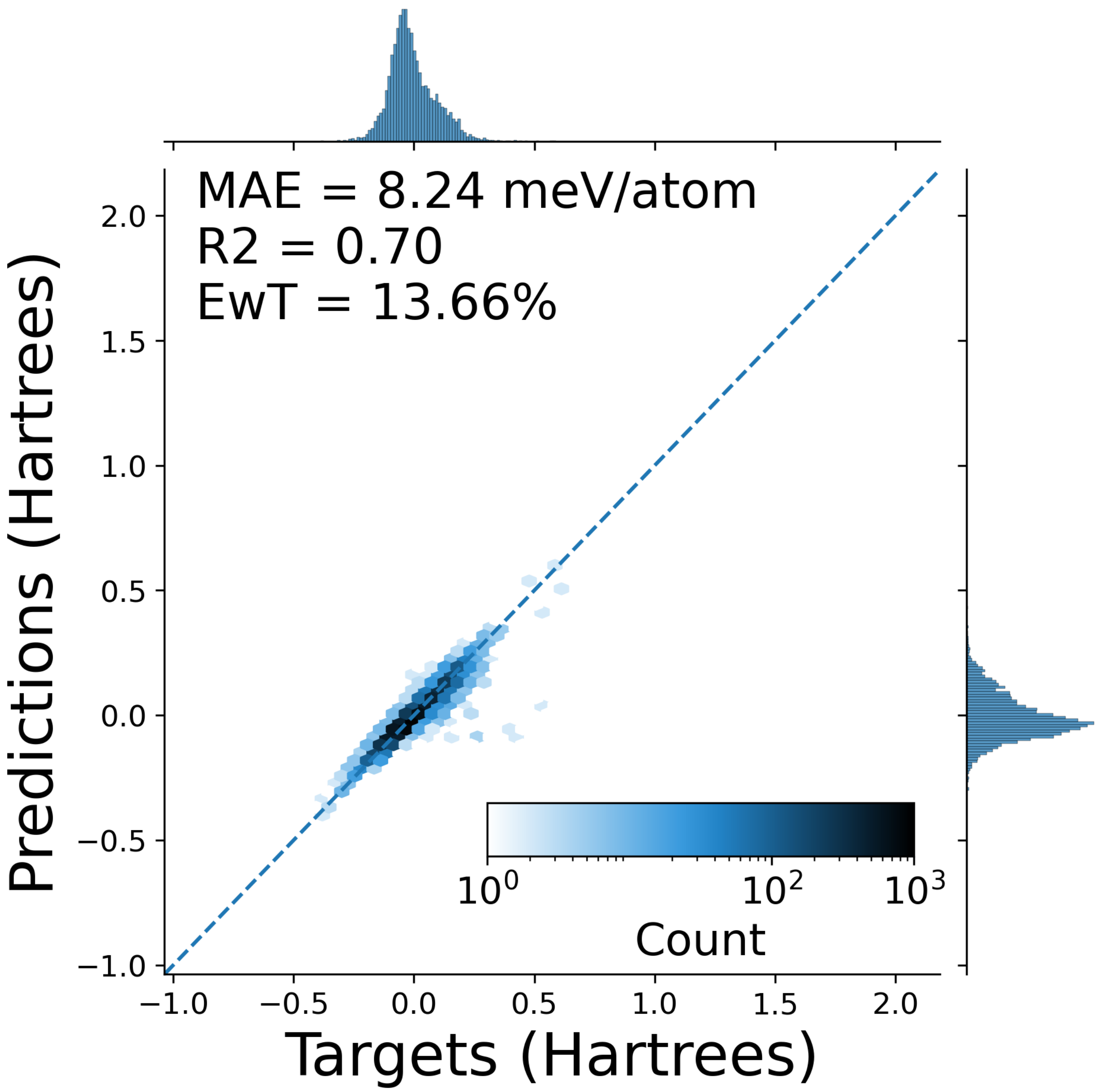

Figure 16: Parity plot for the testing set of a GemNet-T model trained on 80% of all of tmQM, with the datapoints corresponding to structures removed from tmQM to tmQM_wB97MV removed. Of the 8,666 structures in the testing set, 157 structures had an absolute error of at least 0.1 Hartree, and 32 had errors of at least 0.5 Hartree. 23 structures in this set were structures that were removed when generating tmQM_wB97MV.

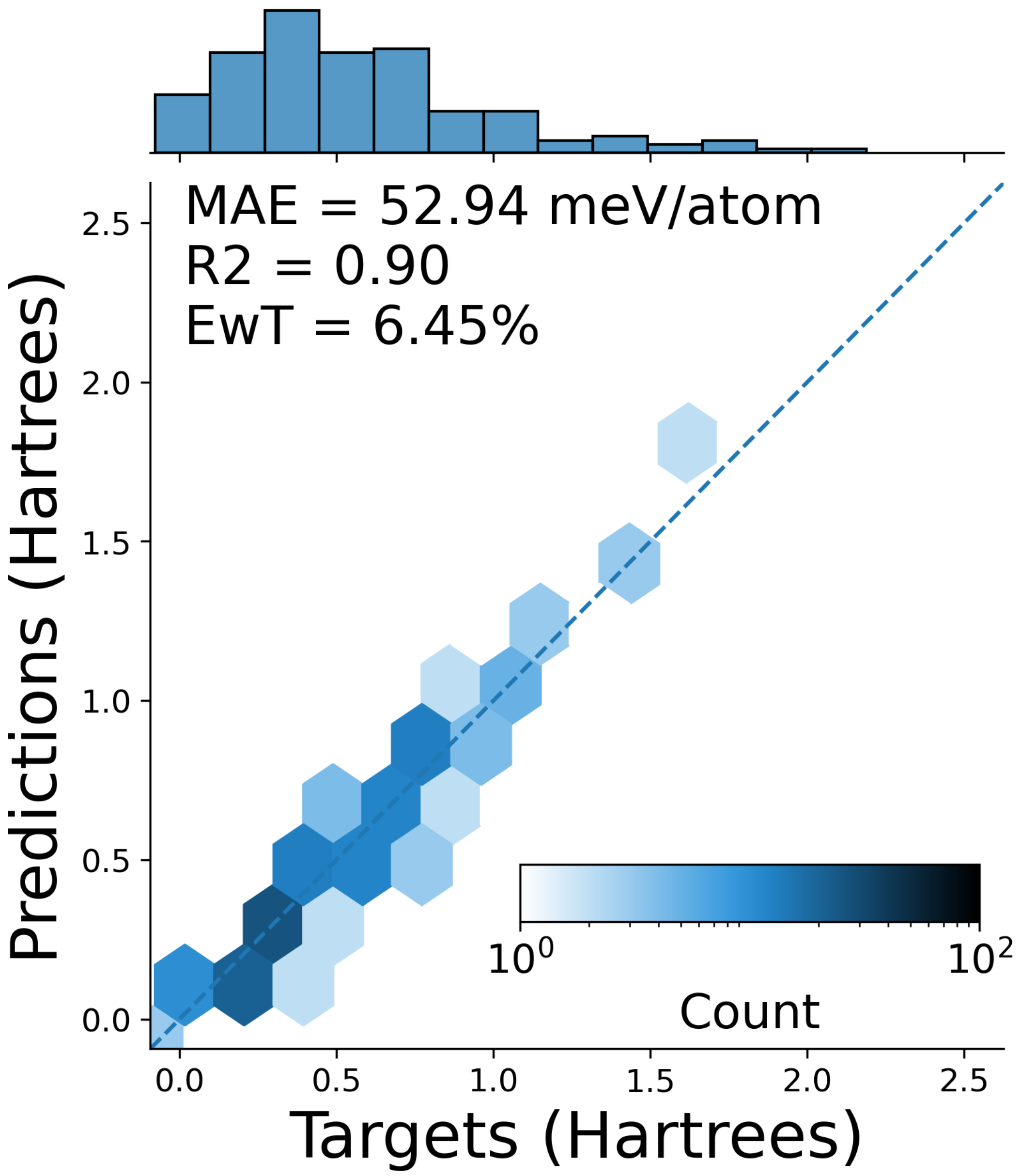

Figure 17: Parity plot for a GemNet-T model trained on 80% of all of tmQM, with only the datapoints corresponding to structures removed from tmQM to tmQM_wB97MV shown (note that this includes structures from across the training, validation, and testing sets). Of the 155 removed structures, 29 had errors of at least 0.1 Hartree, and 1 had errors of at least 0.5 Hartree. We note that of the 155 removed structures, 27 were charged and 128 were neutral, which is a very similar ratio to the ratio of charged to neutral structures in the entire dataset (71,173 neutral structures out of 86,665).

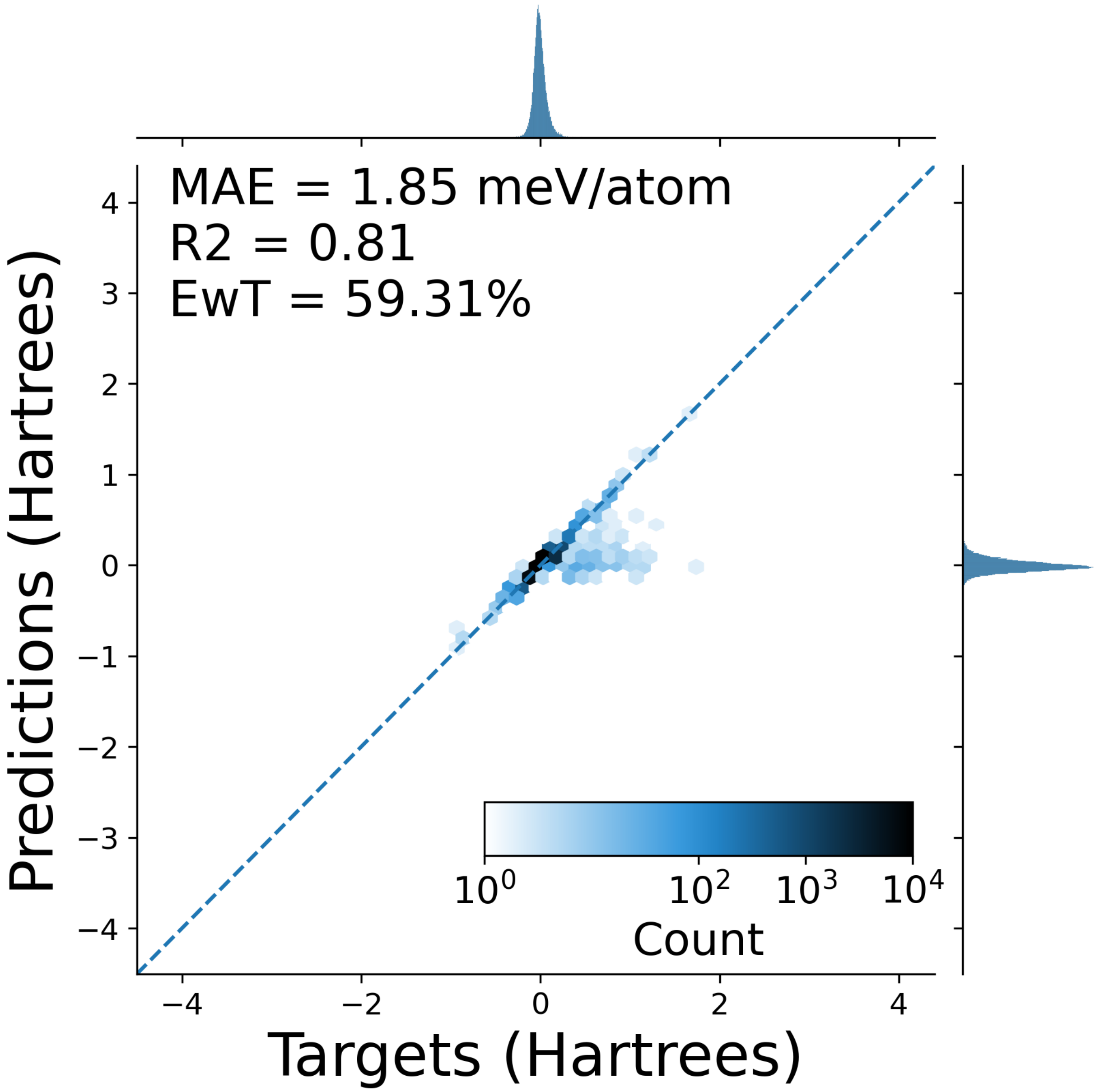

Figure 18: Parity plot for the training set of a GemNet-T model trained on 80% of the neutral subset of tmQM, with the datapoints corresponding to structures removed from tmQM to tmQM_wB97MV removed. Of the 56,939 structures in the training set, 287 structures had an absolute error of at least 0.1 Hartree, and 139 had errors of at least 0.5 Hartree. 104 structures in this set were structures that were removed when generating tmQM_wB97MV.

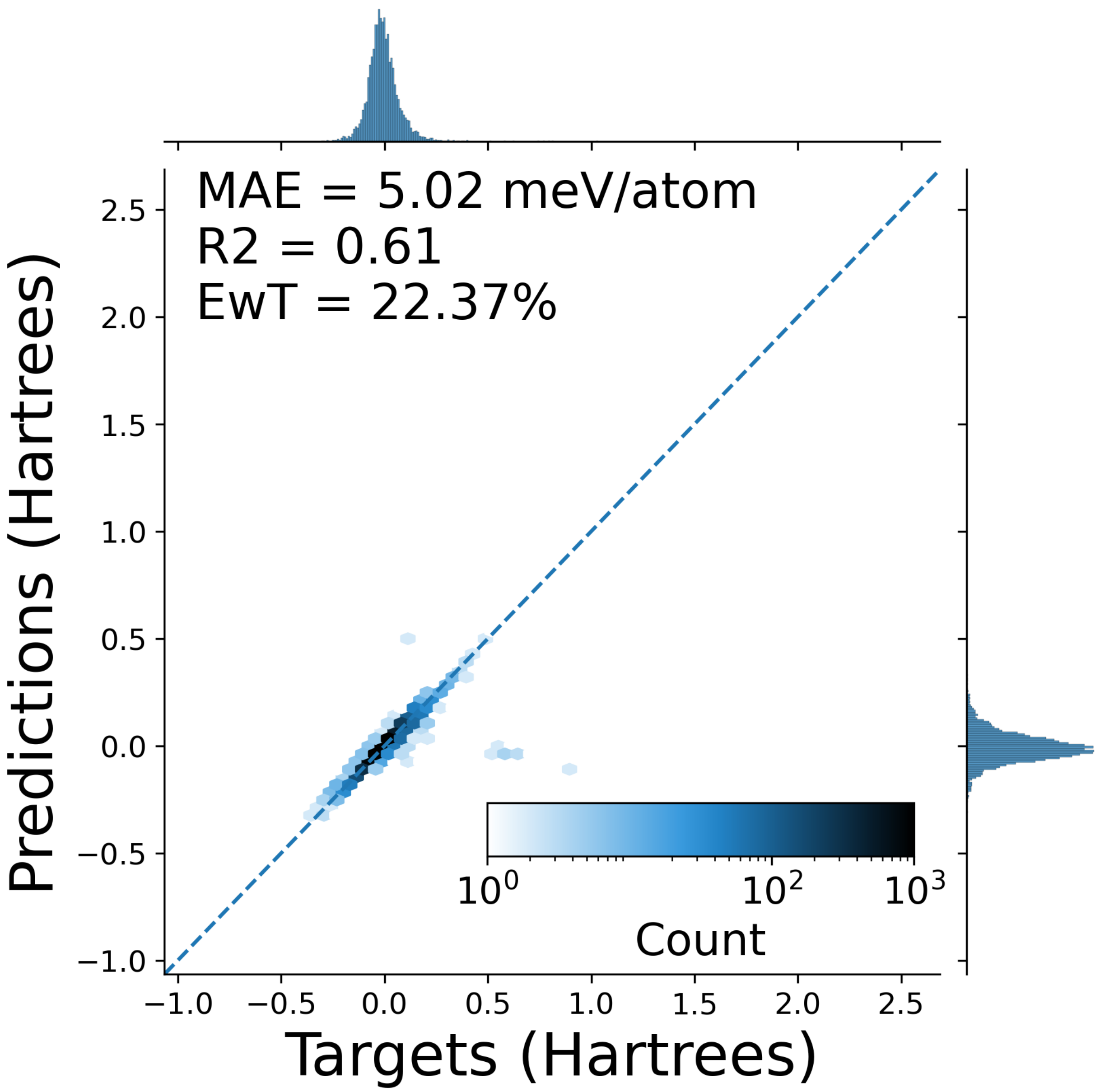

Figure 19: Parity plot for the validation set of a GemNet-T model trained on 80% of the neutral subset of tmQM, with the datapoints corresponding to structures removed from tmQM to tmQM_wB97MV removed. Of the 7,117 structures in the validation set, 78 structures had an absolute error of at least 0.1 Hartree, and 32 had errors of at least 0.5 Hartree. 10 structures in this set were structures that were removed when generating tmQM_wB97MV.

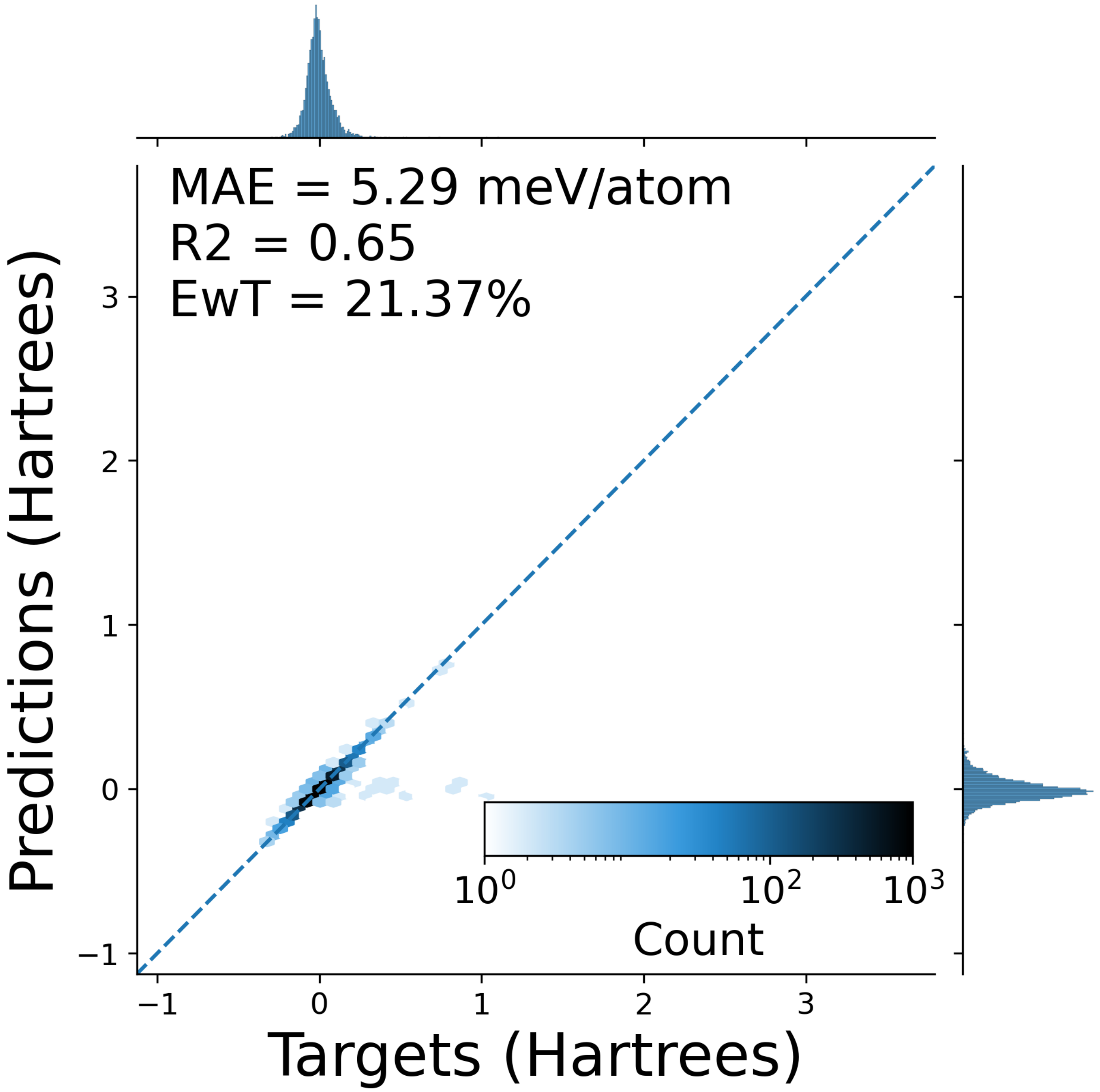

Figure 20: Parity plot for the testing set of a GemNet-T model trained on 80% of the neutral subset of tmQM, with the datapoints corresponding to structures removed from tmQM to tmQM_wB97MV removed. Of the 7,117 structures in the testing set, 72 structures had an absolute error of at least 0.1 Hartree, and 36 had errors of at least 0.5 Hartree. 14 structures in this set were structures that were removed when generating tmQM_wB97MV.

tmQM_wB97MV MAE and EwT Tables

Table 9: Test set Mean Absolute Error (in meV/atom) for all models trained on all of tmQM_wB97MV.

	MAE (meV/atom)			
Training %	SchNet	PaiNN	SpinConv	GemNet-T
20%	23	19	16	**15**
40%	19	13	12	**10**
60%	17	10	10	**8**
80%	16	9	10	**8**

Table 10: Test set Mean Absolute Error (in meV/atom) for models trained on the neutral subset of tmQM_wB97MV.

	MAE (meV/atom)			
Training %	SchNet	PaiNN	Spinconv	GemNet-T
20%	11	9	8	**7**
40%	9	7	6	**5**
60%	8	6	5	**4**
80%	8	6	5	**4**

Table 11: Test set Energy within Threshold (EwT, %) for models trained on the neutral subset of tmQM_wB97MV.

	Energy within Threshold (EwT, %)			
Training %	SchNet	PaiNN	Spinconv	GemNet-T
20%	5.8	7.6	8.9	**9.8**
40%	7.9	10.3	12.1	**15.7**
60%	8.8	13.0	14.3	**21.0**
80%	10.5	15.3	14.3	**25.2**

tmQM_wB97MV Test Parity Plots

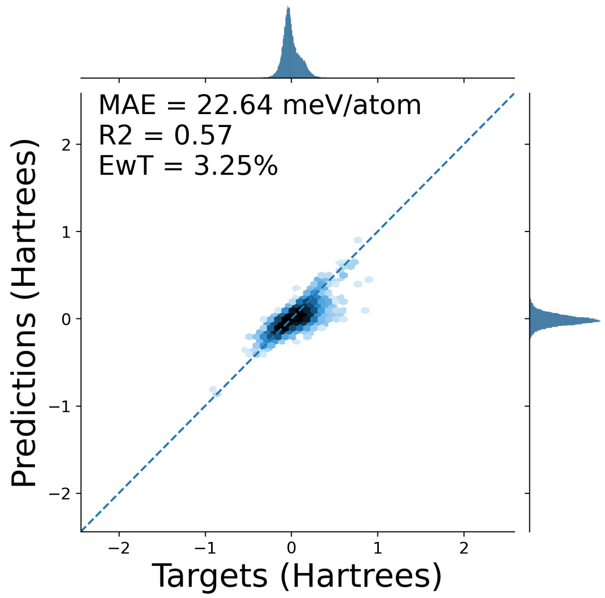

(a) SchNet

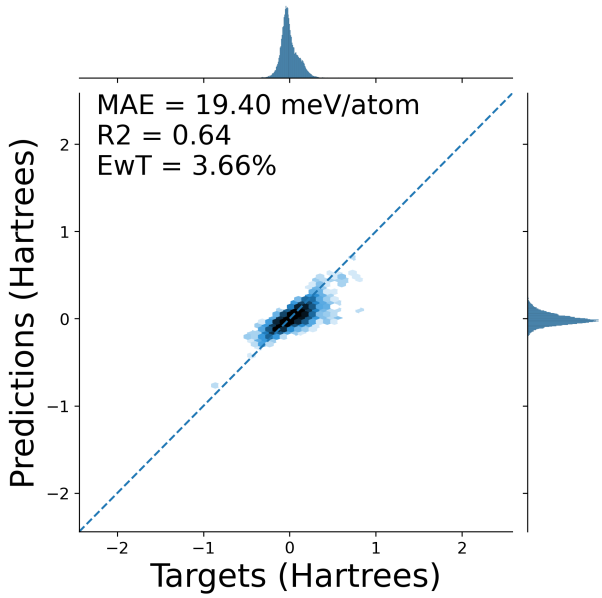

(b) PaiNN

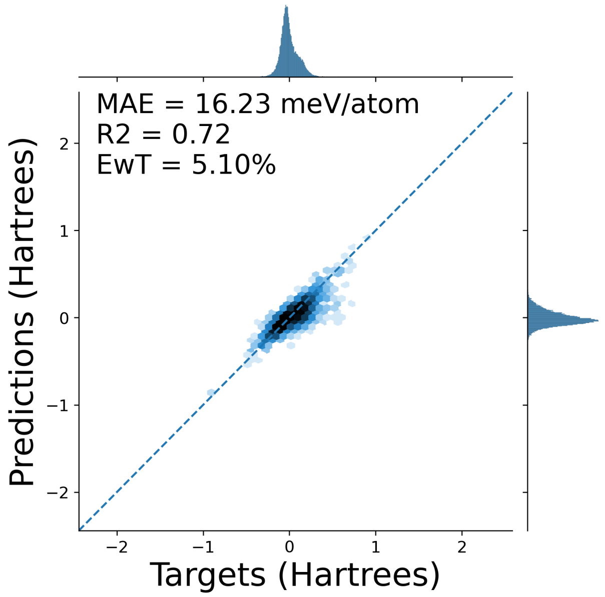

(c) SpinConv

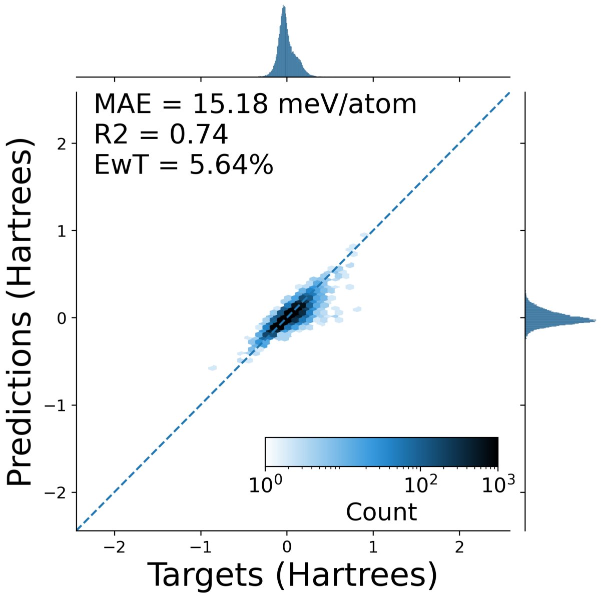

(d) GemNet-T

Figure 21: Parity plots for the test set of models trained on 20% of tmQM_wB97MV.

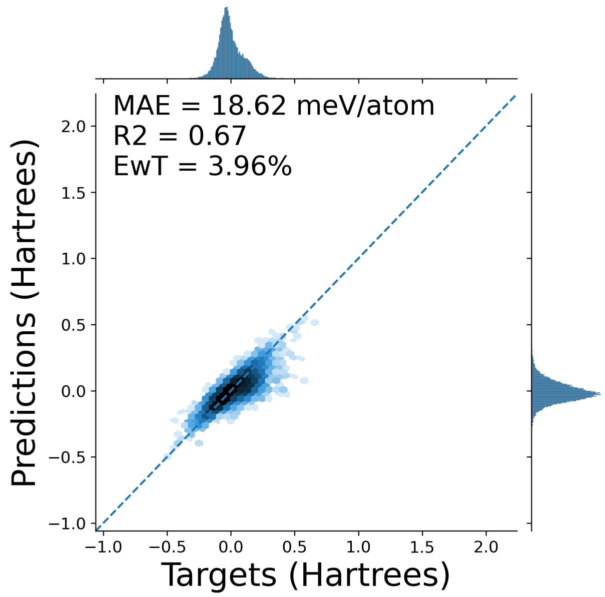

(a) SchNet

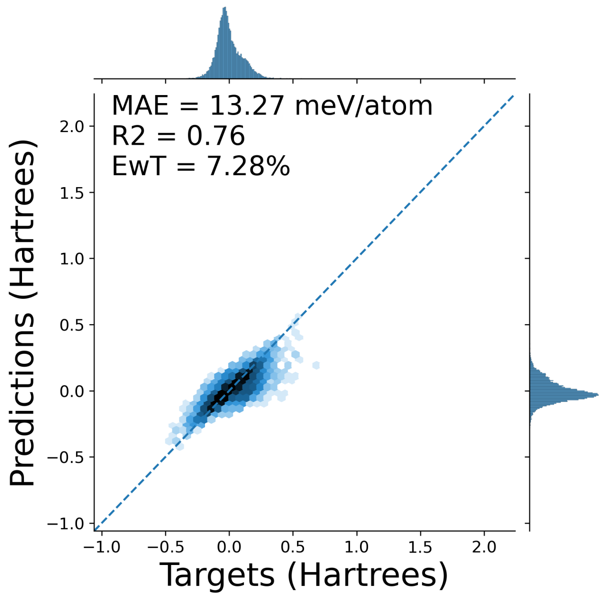

(b) PaiNN

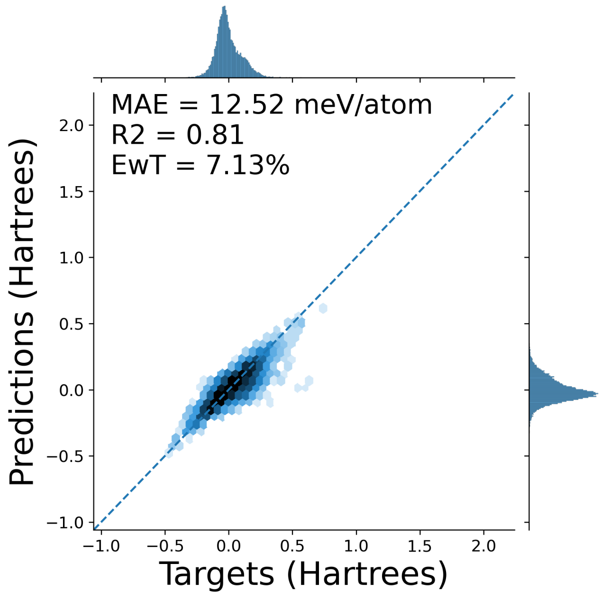

(c) SpinConv

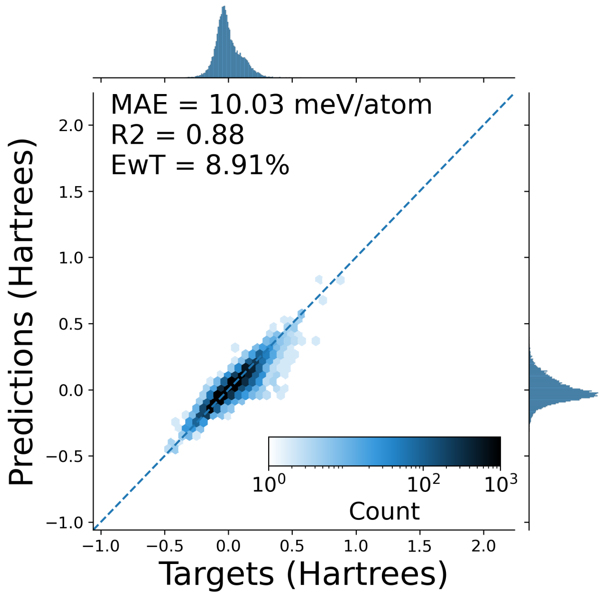

(d) GemNet-T

Figure 22: Parity plots for the test set of models trained on 40% of tmQM_wB97MV.

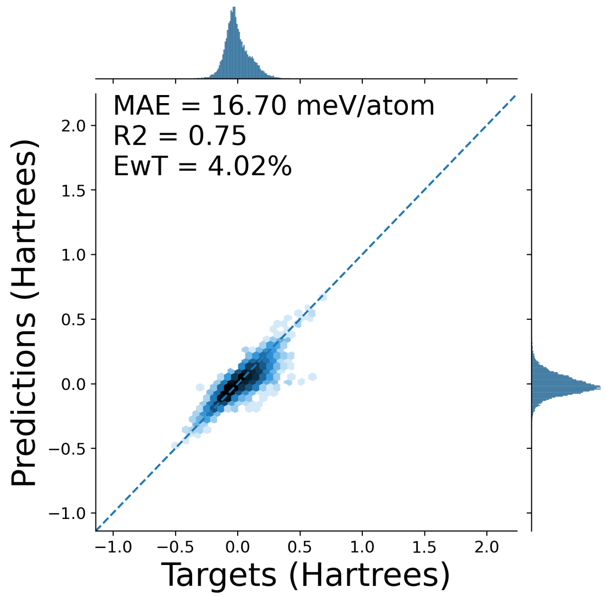

(a) SchNet

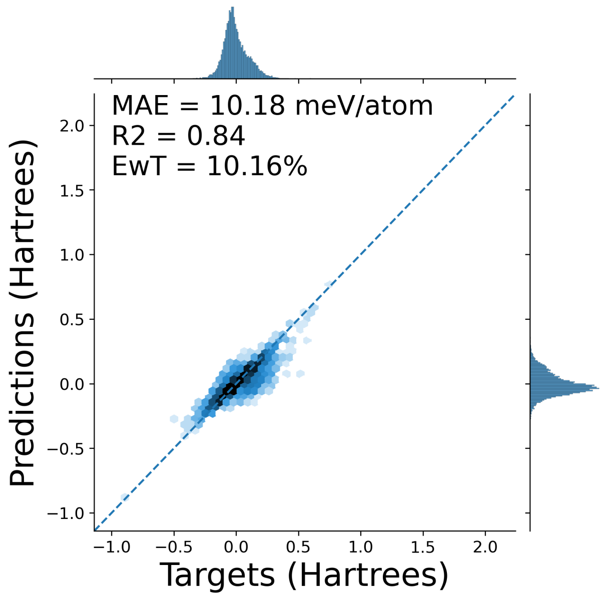

(b) PaiNN

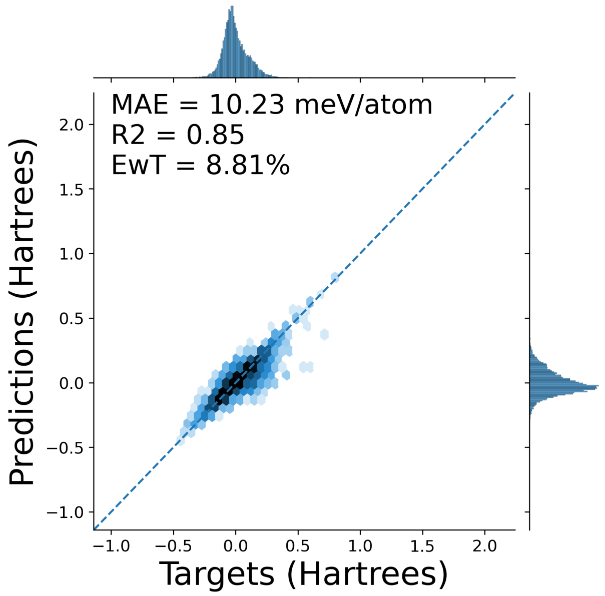

(c) SpinConv

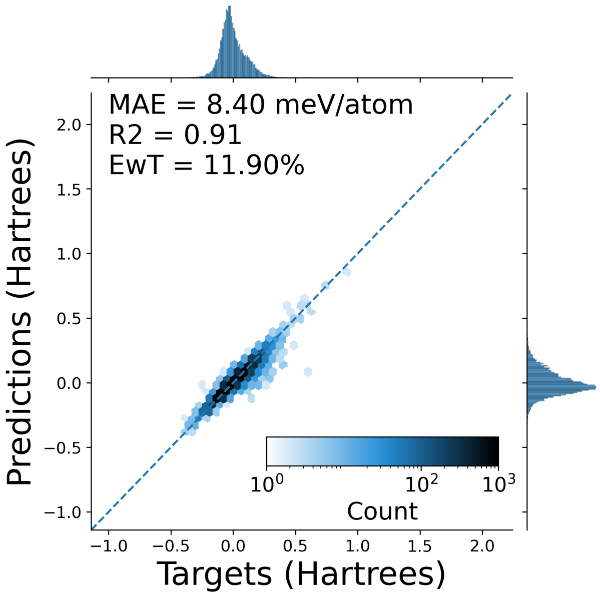

(d) GemNet-T

Figure 23: Parity plots for the test set of models trained on 60% of tmQM_wB97MV.

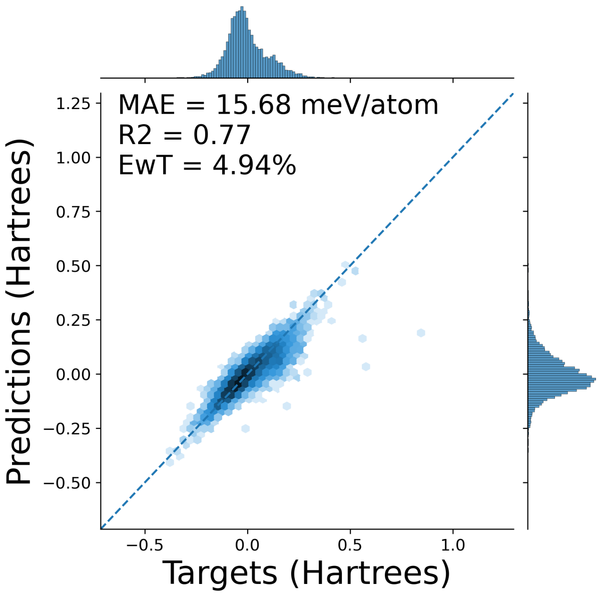

(a) SchNet

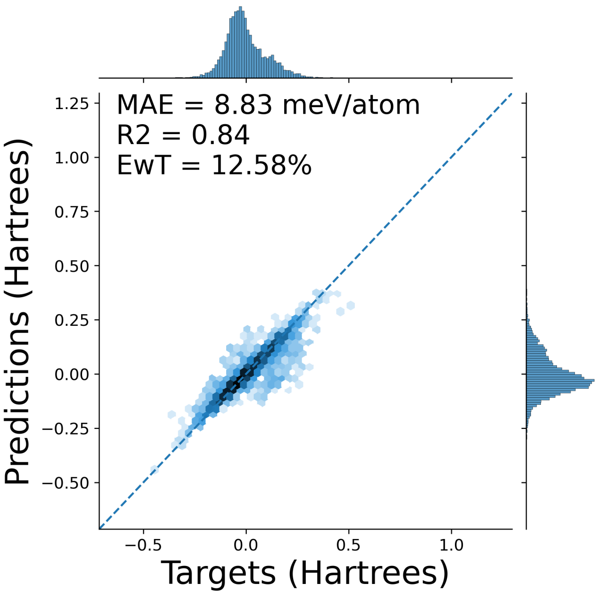

(b) PaiNN

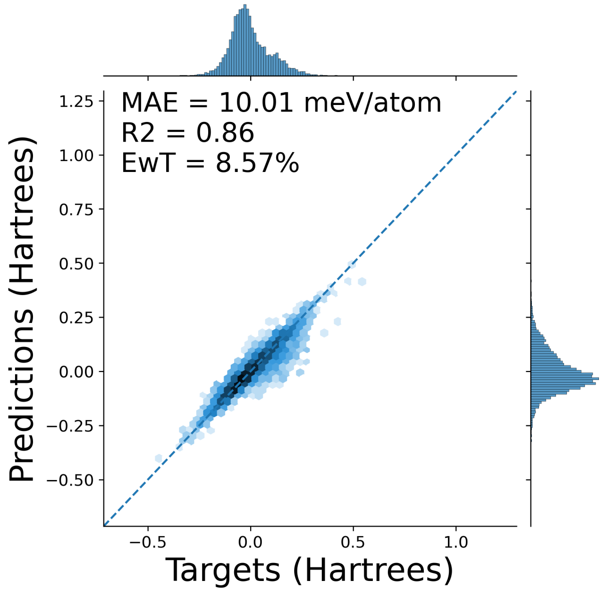

(c) SpinConv

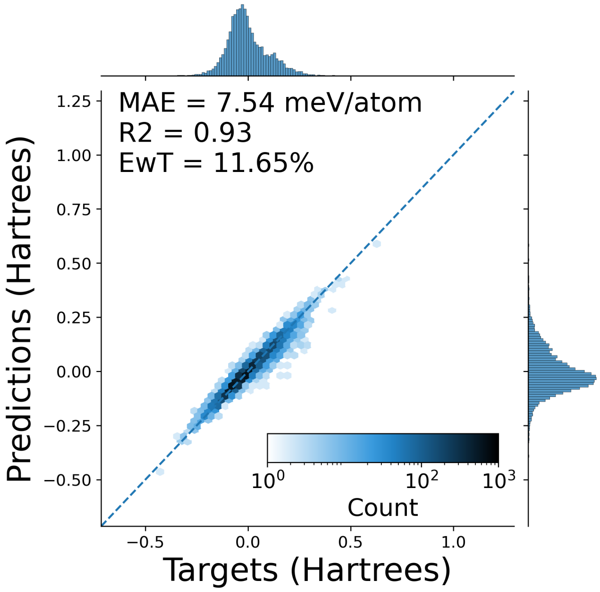

(d) GemNet-T

Figure 24: Parity plots for the test set of models trained on 80% of tmQM_wB97MV.

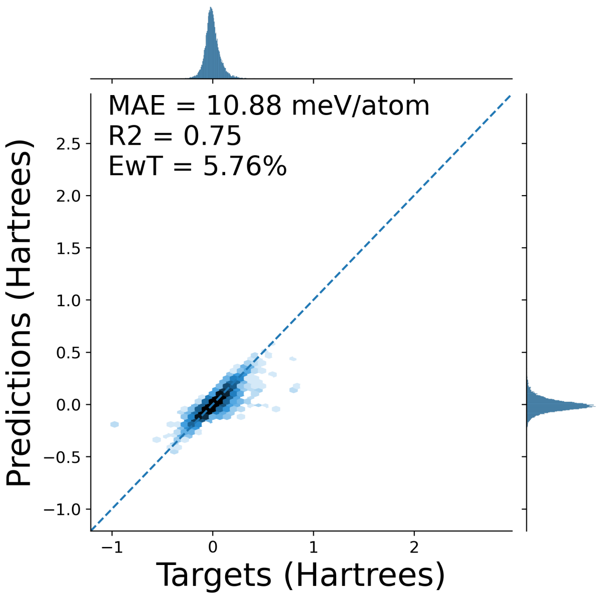

(a) SchNet

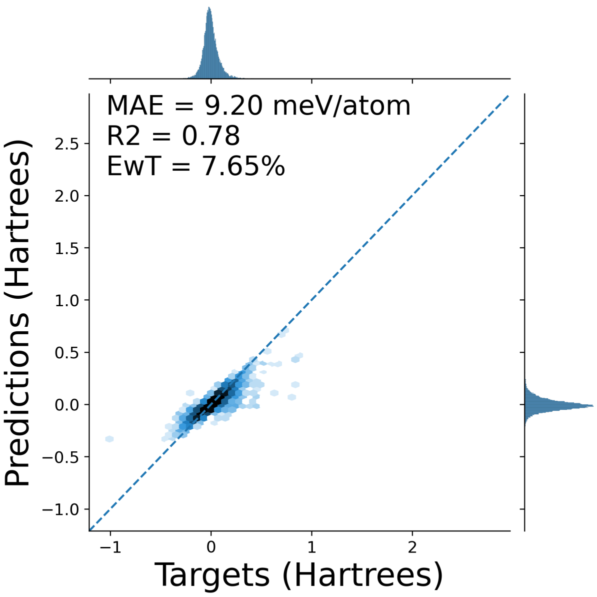

(b) PaiNN

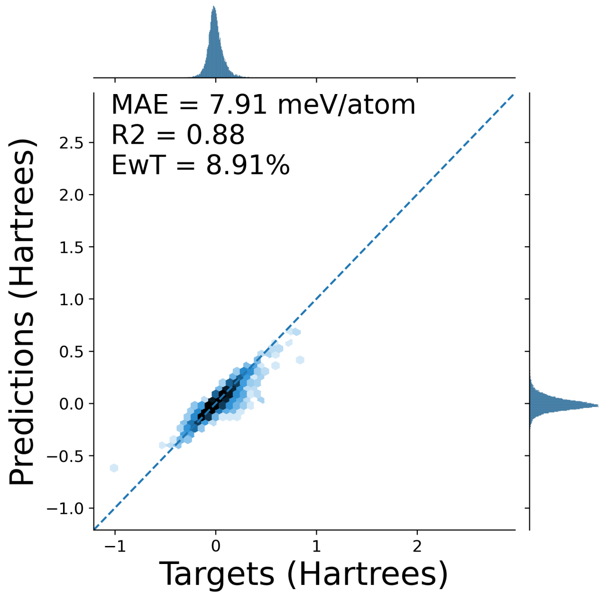

(c) SpinConv

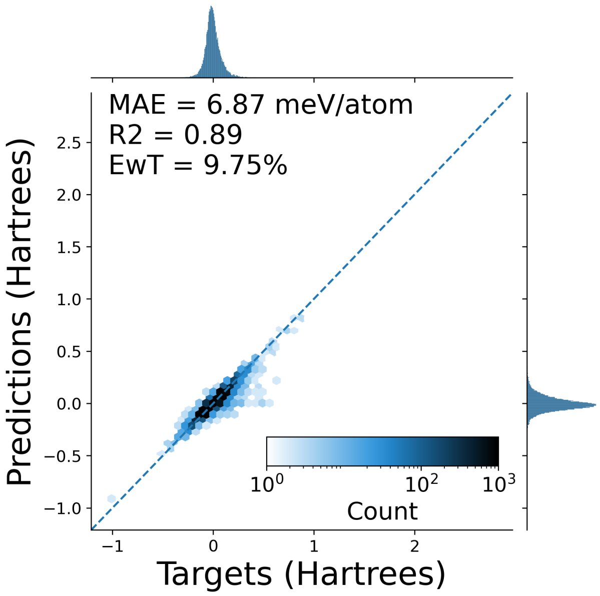

(d) GemNet-T

Figure 25: Parity plots for the test set of models trained on 20% of the neutral subset of tmQM_wB97MV.

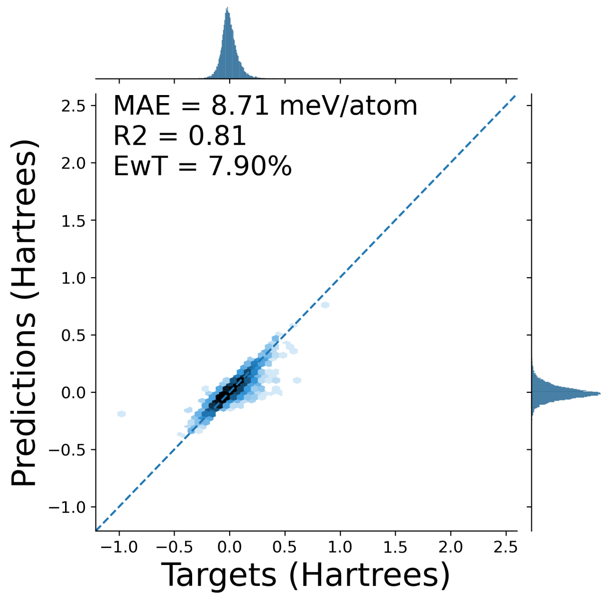

(a) SchNet

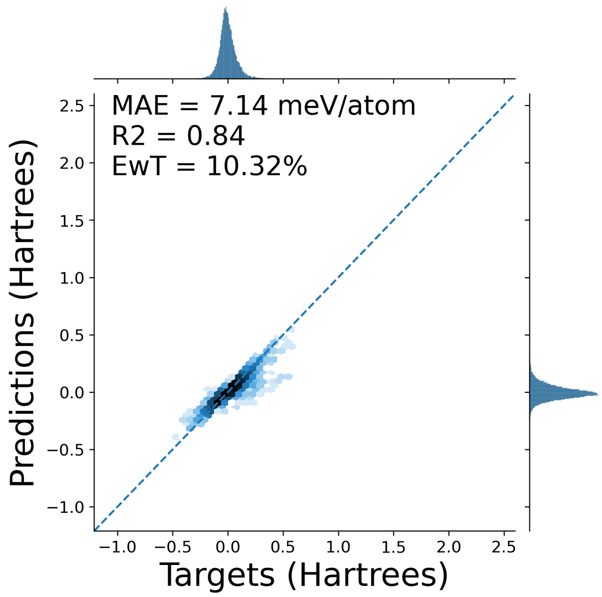

(b) PaiNN

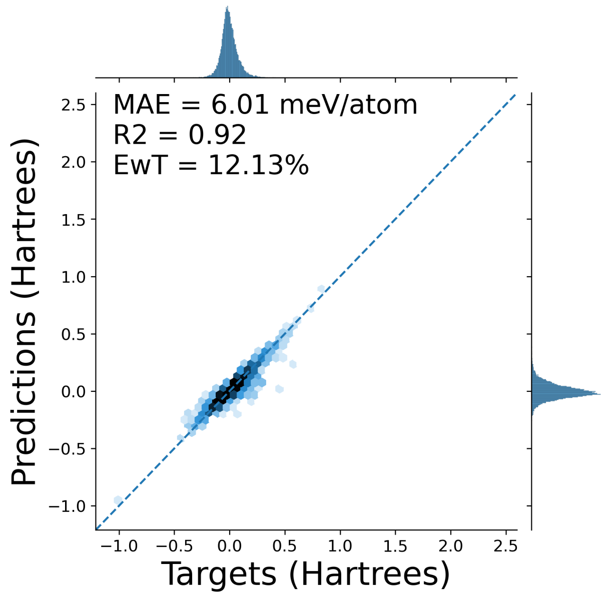

(c) SpinConv

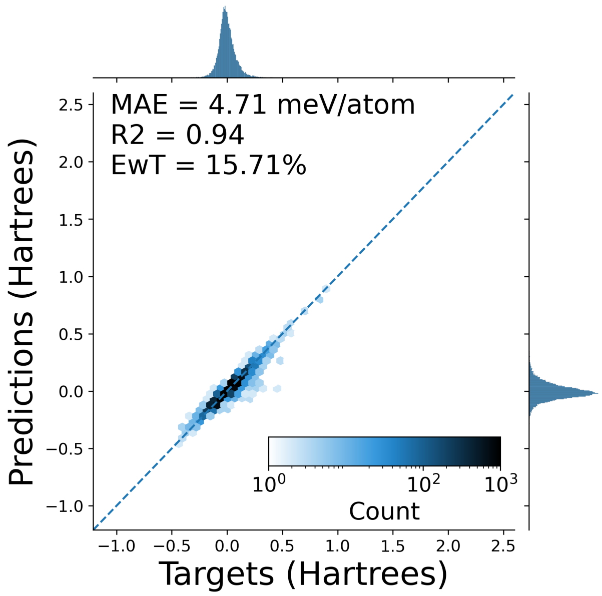

(d) GemNet-T

Figure 26: Parity plots for the test set of models trained on 40% of the neutral subset of tmQM_wB97MV.

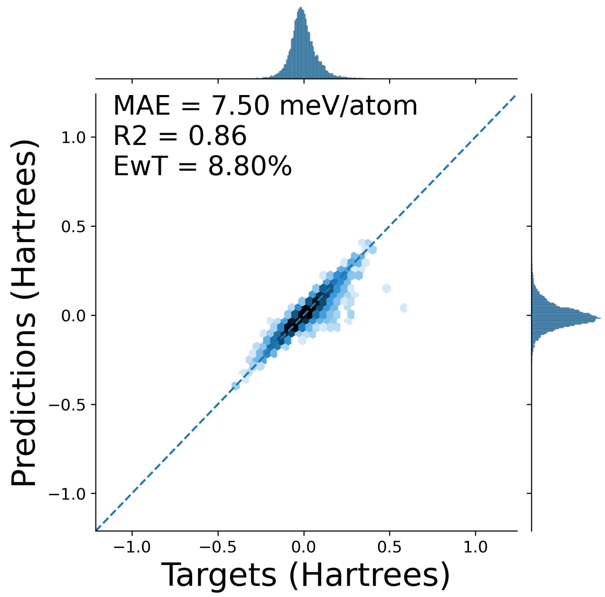

(a) SchNet

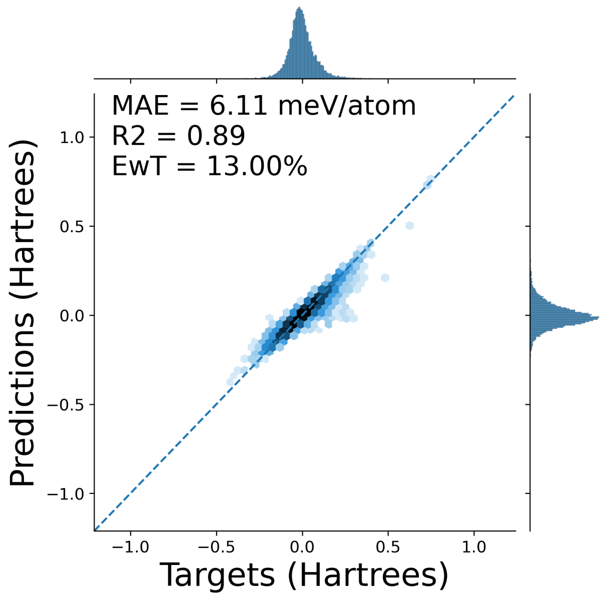

(b) PaiNN

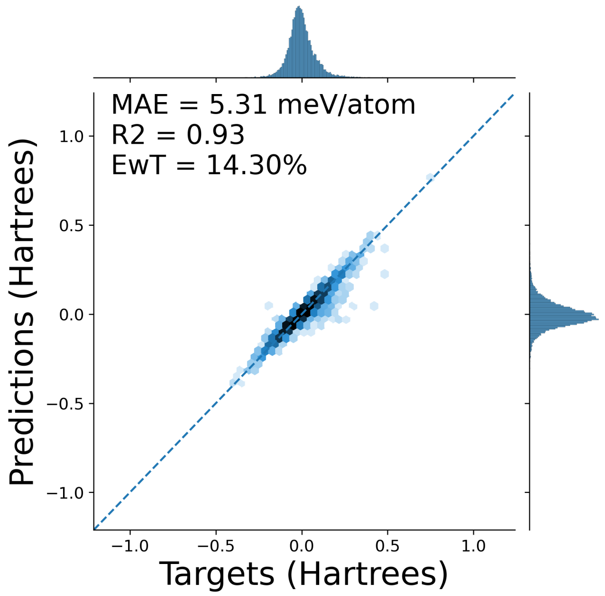

(c) SpinConv

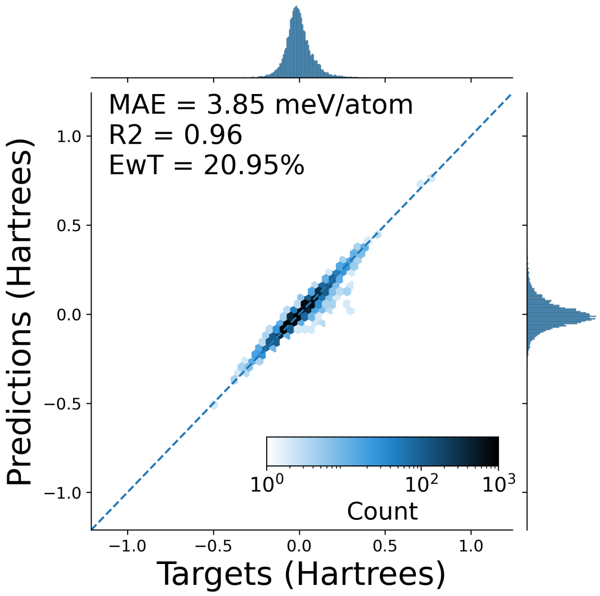

(d) GemNet-T

Figure 27: Parity plots for the test set of models trained on 60% of the neutral subset of tmQM_wB97MV.

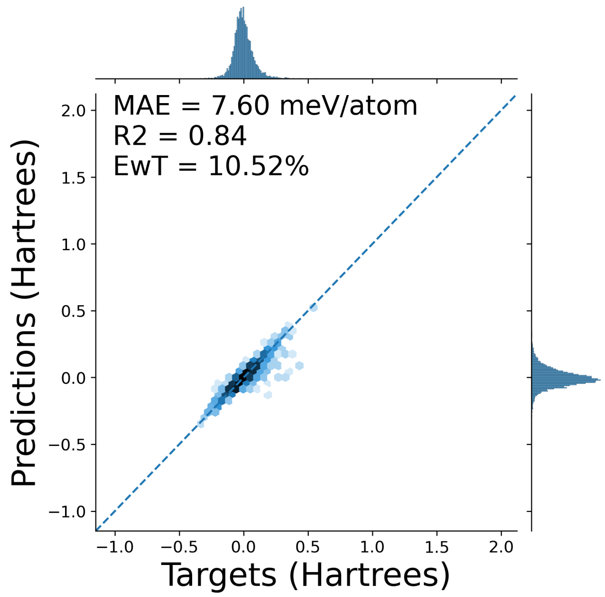

(a) SchNet

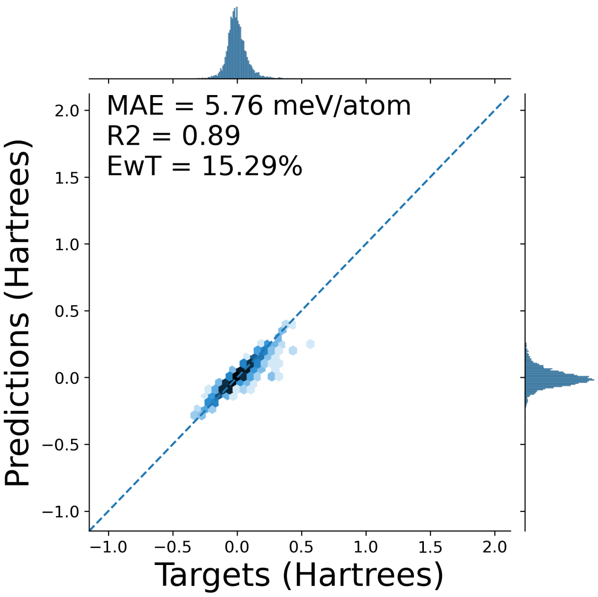

(b) PaiNN

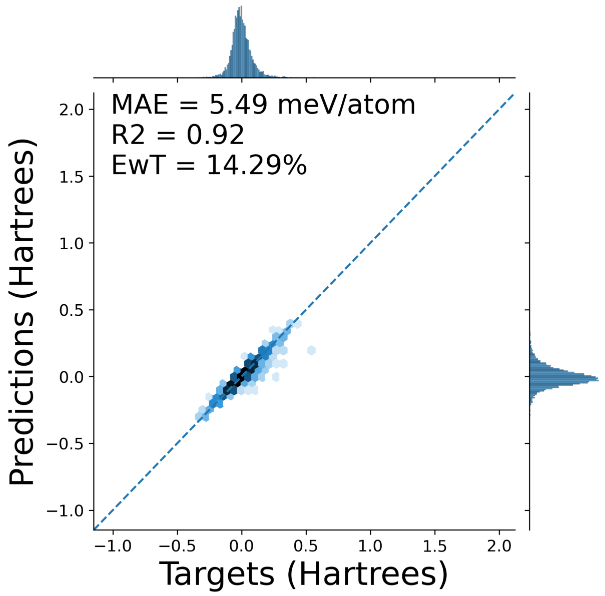

(c) SpinConv

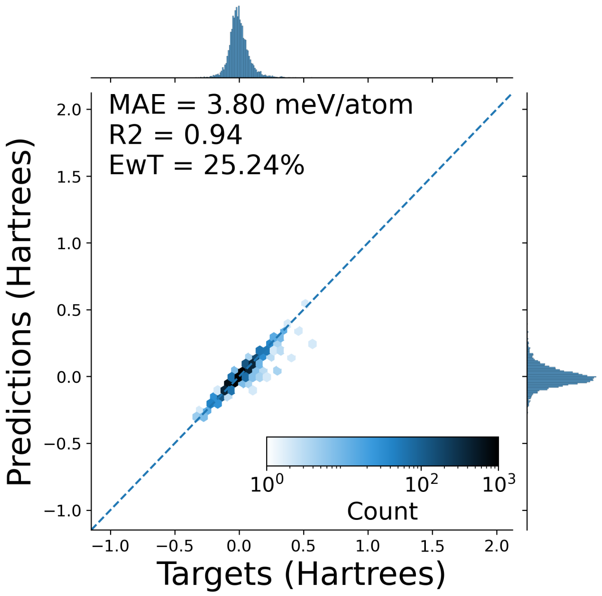

(d) GemNet-T

Figure 28: Parity plots for the test set of models trained on 80% of the neutral subset of tmQM_wB97MV.
